# Inhibiting ERK5 Overcomes Breast Cancer Resistance to Anti-HER2 Therapy By Targeting the G_1_–S Cell-Cycle Transition

**DOI:** 10.1158/2767-9764.CRC-21-0089

**Published:** 2022-03-10

**Authors:** Jingwei Zhang, Adam J. Pearson, Nitin Sabherwal, Brian A. Telfer, Nisha Ali, Karmern Kan, Qiuping Xu, Wei Zhang, Fuhui Chen, Shiyang Li, Jinhua Wang, Nathanael S. Gray, Blanca Risa-Ebrí, Katherine G. Finegan, Michael J. Cross, Emanuele Giurisato, Alan J. Whitmarsh, Cathy Tournier

**Affiliations:** 1 Division of Cancer Sciences, School of Medical Sciences, Faculty of Biology, Medicine and Health (FBMH), University of Manchester, Manchester, United Kingdom.; 2 Division of Developmental Biology and Medicine, School of Medical Sciences, FBMH, University of Manchester, Manchester, United Kingdom.; 3 Division of Pharmacy and Optometry, School of Health Sciences, FBMH, University of Manchester, United Kingdom.; 4 Manchester University NHS FT, Wythenshawe Hospital, Manchester, United Kingdom.; 5 Guangdong Provincial Key Laboratory of Malignant Tumor Epigenetics and Gene Regulation, Sun Yat-Sen Memorial Hospital, Sun Yat-Sen University, Guangzhou 510120, China.; 6 Wellcome Centre for Cell-Matrix Research, School of Biological Sciences, FBMH, University of Manchester, Manchester, United Kingdom.; 7 Department of Cancer Biology, Dana-Farber Cancer Institute, and Department of Biological Chemistry and Molecular Pharmacology, Harvard Medical School, Boston, Massachusetts.; 8 Stanford Cancer Institute, Chem-H, Chemical and Systems Biology, Stanford University, Stanford, CA 94305.; 9 Department of Pharmacology and Therapeutics, Institute of Systems, Molecular and Integrative Biology (ISMIB), University of Liverpool, Liverpool, United Kingdom.; 10 Department of Biotechnology Chemistry and Pharmacy, University of Siena, Siena, Italy.; 11 Division of Molecular and Cellular Function, School of Biological Sciences, FBMH, University of Manchester, Manchester, United Kingdom.

## Abstract

Targeting HER2 has become a landmark in the treatment of HER2-driven breast cancer. Nonetheless, the clinical efficacy of anti-HER2 therapies can be short-lived and a significant proportion of patients ultimately develop metastatic disease and die. One striking consequence of oncogenic activation of HER2 in breast cancer cells is the constitutive activation of the extracellular-regulated protein kinase 5 (ERK5) through its hyperphosphorylation. In this study, we sought to decipher the significance of this unique molecular signature in promoting therapeutic resistance to anti-HER2 agents. We found that a small-molecule inhibitor of ERK5 suppressed the phosphorylation of the retinoblastoma protein (RB) in HER2-positive breast cancer cells. As a result, ERK5 inhibition enhanced the antiproliferative activity of single-agent anti-HER2 therapy in resistant breast cancer cell lines by causing a G_1_ cell-cycle arrest. Moreover, ERK5 knockdown restored the antitumor activity of the anti-HER2 agent lapatinib in human breast cancer xenografts. Taken together, these findings support the therapeutic potential of ERK5 inhibitors to improve the clinical benefit that patients receive from targeted HER2 therapies.

**Significance:**

Here we demonstrate that targeting ERK5 in HER2-positive breast cancer cells reduces the level of phosphorylation of RB, an important mediator of the G_1_–S transition. This effect is associated with increased antitumor activity of lapatinib in combination therapy with ERK5 silencing. Collectively, these findings reveal that ERK5 constitutes a relevant therapeutic target for the many patients with resistant HER2-positive breast cancer.

## Introduction

HER2, also known as erb-b2 receptor tyrosine kinase 2 (ERBB2), is a transmembrane tyrosine kinase receptor that is overexpressed in around 20% of all breast cancers, leading to a poor prognosis for patients (1, 2). HER2 stimulation following homodimerization or heterodimerization with other members of the EGFR family promotes tumor growth and metastasis principally through activation of the MAPK and PI3K/AKT/mTOR pathways (3). Accordingly, targeting HER2 has markedly improved the outcome for patients with HER2-positive (+) breast cancer (4). The recommended first-line HER2-targeting agents are the mAbs trastuzumab (Herceptin) and pertuzumab (Perjeta) that specifically bind to the extracellular domain of HER2. After the first-line treatment has failed, the second-line antibody–drug conjugate trastuzumab emtansine (Kadcyla)-based therapy is utilized in the adjuvant/postadjuvant setting. Current practices in third-line treatments typically include small-molecule tyrosine kinase inhibitors, for example, lapatinib (TyveRB), neratinib (Nerlynx), and tucatinib (Tukysa). Despite the survival gains provided by HER2-targeted therapies, a high mortality rate persists as intrinsic or acquired resistance frequently occurs (5, 6). The magnitude of this clinical challenge calls for continued research to understand how breast cancer cells escape the antitumor activity of HER2-targeting agents.

The best-characterized mechanism that confers resistance to trastuzumab and lapatinib involves the hyperactivation of the PI3K pathway through inactivation of the phosphatase PTEN or activating mutations of the *PIK3CA* locus (encoding the catalytic subunit of PI3K; refs. 7–13). Accordingly, a phase III clinical trial (BOLERO-3) demonstrated that the addition of an inhibitor of mTOR (everolimus), a key downstream effector of PI3K, improved treatment with trastuzumab plus chemotherapy [median progression-free survival (PFS) 7 months with everolimus versus 5.8 months with placebo; ref. 14]. However, patients with HER2^+^ breast cancer whose disease had progressed on a trastuzumab-based therapy did not benefit from subsequently receiving an inhibitor of PI3K (buparlisib) in combination with trastuzumab (15). More recently, several clinical trials to test the efficacy of combined CDK4/6 inhibitors and anti-HER2 agents have been initiated (16, 17). This strategy was based on a clear synergy between anti-HER2 therapy and CDK4/6 inhibitors in HER2^+^ breast cancer cell lines (18, 19) and the demonstration in transgenic mice that the activation of cyclin D1–CDK4/6 was involved in mediating resistance to anti-HER2 therapy (20). Initial findings from the monarcHER phase II trial indicate that the CDK4/6 inhibitor abemaciclib (Verzenios), when combined with trastuzumab and fulvestrant, increased the PFS of patients with HER2^+^ metastatic breast cancer compared with those receiving trastuzumab and standard chemotherapy (median PFS, 8.3 months vs. 5.7 months; ref. 21). The demonstration of efficacy in the phase III PATINA trial (NCT02947685) of palbociclib (Ibrance) in combination with anti-HER2 therapy plus endocrine therapy would lead to the first CDK4/6 inhibitor to be approved for the treatment of hormone receptor–positive, HER2^+^ metastatic breast cancer.

Multiple compensatory pathways ultimately limit the durability of most targeted therapies. Consequently, sequential combinations of therapies and/or their intermittent use might be necessary to prevent resistance. In line with this argument, we sought to identify additional mechanisms responsible for the poor clinical outcome in HER2^+^ breast cancer. Specifically, we explored the role of the extracellular-regulated protein kinase 5 (ERK5), also known as mitogen-activated protein kinase 7 (MAPK7), a nonredundant MAPK with a catalytic core most similar to the classical ERK1/ERK2 subfamily. Unlike the other MAPKs, ERK5 exhibits a unique extended C-terminal tail that becomes hyperphosphorylated upon ERK5 activation by a distinct MAPK/ERK kinase, namely MEK5/MAP2K5 (22, 23). Importantly, ERK5 was previously observed to be constitutively activated in breast cancer cells overexpressing HER2 (24). Moreover, genetic inactivation of ERK5 was shown to enhance the antiproliferative effect of trastuzumab in HER2^+^ breast cancer cells (25). In this study, we demonstrate that inhibition of ERK5 is a feasible approach to overcome resistance to anti-HER2 agents and propose ERK5 as a promising novel therapeutic target to increase the antitumor action of existing HER2-blocking strategies for treating breast cancer.

## Materials and Methods

### Animal Welfare and Human Samples

Mice were maintained in a pathogen-free facility at the University of Manchester (Manchester, United Kingdom). All animal procedures were performed under license in accordance with the UK Home Office Animals (Scientific Procedures) Act (1986) and approved by the Animal Welfare and Ethical Review Body of the University of Manchester (Manchester, United Kingdom). In particular, mice with tumors were closely monitored daily for any changes in their overall conditions. Human breast tissues were obtained from the Manchester Cancer Research Centre (MCRC) Biobank, with written informed consent from the patients. The MCRC Biobank holds a generic ethics approval which can confer this approval to users of banked samples via the MCRC Biobank Access Policy. The role of the MCRC biobank is to distribute research samples and therefore, cannot endorse studies performed or the interpretation of results.

### Cell Lines, Cell Culture, and Reagents

Human breast cancer cell lines BT474, SK-BR-3, MDA-MB-361, and MDA-MB-453 were purchased from the ATCC at the beginning of the project in August 2018. Cell lines were cultured in DMEM (Sigma #D6429) supplemented with 10% FBS (Sigma #10500–064) and 1% penicillin/streptomycin (Sigma #P4333), except MDA-MB-361 cells which were grown in DMEM supplemented with 10% FBS, 1% penicillin/streptomycin, 2 mmol/L l-glutamine (Sigma #G7513), and 1× MEM nonessential amino acid solution (Sigma #M7145). Lapatinib-resistant (LR) and trastuzumab-resistant (TR) BT474 cell lines were developed by incubation with gradually increased doses of lapatinib (APExBIO #A8218) or trastuzumab (Genentech) over a period of 4 to 5 months. Resistant cells were subsequently maintained with 0.1 μmol/L lapatinib or 50 μg/mL trastuzumab. The compounds were removed from the culture medium three to four days prior to experiments. Cell authentication was not routinely conducted given that cell lines were utilized for a maximum of 20 passages before thawing another aliquot of the same stock to maintain the original phenotype. *Mycoplasma* testing was not routinely performed on the cells. Trametinib was purchased from APExBIO (#A3887). For *in vivo* studies, lapatinib was dissolved in a buffer containing 1% Tween-80 and 5% hydroxypropyl methylcellulose just before use.

### Histological and Immunohistochemical (IHC) Staining

For histological analysis, sections were stained with hematoxylin and eosin (H&E). For IHC, formalin-fixed paraffin-embedded human and murine sections were deparaffinized with xylene, and rehydrated using ethanol at 100%, 95%, and 75%. After washing in water, the slides were microwaved for 15 minutes in EDTA buffer (pH = 6) for antigen retrieval. Endogenous peroxidase activity was blocked with 0.3% hydrogen peroxidase for 15 minutes at room temperature. Slides were incubated with an antibody to ERK5 (1:100 dilution; Abcam #ab196609) or to Ki67 (1:200 dilution; Abcam #ab16667) overnight at 4°C. The antigen was revealed using diaminobenzidine (DAB, brown) peroxidase substrate kit (Vector Laboratories, #SK-4100) and counterstained with hematoxylin (blue). H&E and immunostained slides with the ERK5 antibody were examined by a pathologist.

### Protein Extraction and Immunoblot Analysis

Proteins were extracted from cells in RIPA buffer (Sigma-Aldrich# R0278) supplemented with protease and phosphatase inhibitors. Protein concentrations were quantified by DC protein assay (Bio-Rad #500–0113/0114/0115). Extracts (25 μg) were resolved by SDS-PAGE and subjected to immunoblot analysis with the following antibodies from Cell Signaling Technology: HER2 (#2165), pHER2 (Y1221/1222; #2243), ERK5 (#3372), ERK1/2 (#9102), pERK1/2 (#4370), RB (#9309), pRB (S780; #8180), S6RP (#2217), pS6RP (S235/236; #4858), or β-tubulin (#5346). Immunecomplexes were detected by enhanced chemiluminescence with immunoglobulin G coupled to horseradish peroxidase as the secondary antibody (GE Healthcare #NA931 and #NA934).

### Crystal Violet Staining and Cell-Cycle Analysis

Breast cancer cells were seeded in duplicate at 200,000 (BT474, BT474-LR and BT474-TR), 30,000 (SK-BR-3), 80,000 (MDA-MB-361), or 50,000 (MDA-MB-453) cells per well in 12-well plates to allow proliferation in FBS-containing medium. Inhibitors were added the day after plating and the medium containing inhibitors was changed every other day until the experiment ended. The number of adherent cells was estimated by optical density (OD) at 590 nm after fixation in methanol and staining with crystal violet (Sigma-Aldrich). For cell-cycle analyses, cells in log phase growth were treated with the inhibitors and harvested after 24 hours. Cell were subsequently washed twice in PBS, fixed in ice-cold 70% ethanol, and stored at −20°C for at least 24 hours. Fixed cells were incubated in 500 μL of propidium iodide (PI)/RNAse staining solution (Cell Signaling Technology #4087) according to the manufacturer's instructions. The cells were then resuspended in 400 μL of DPBS for flow analysis. All experiments were performed in duplicate.

### EdU Labeling and Staining

Breast cancer cells grown on glass coverslips were stained with 10 μmol/L EdU (Click-iT Edu Alexa Fluor; Thermo Fisher Scientific #C10337) according to the manufacturer's instructions. After staining, cells were washed several times with PBS containing 0.5% Triton X-100 and mounted with ProLong Gold antifade with DAPI (Molecular Probes). Fluorescent cells were viewed with a Leica DM5000 B fluorescence microscope. Images were analyzed using ImageJ software.

### Live-imaging of mCherry-PNCA Based Cell-Cycle Reporter

MDA-MB-453 cells expressing the mCherry-PCNA–based cell cycle reporter were seeded overnight in 8-well μ-slide imaging polymer dishes coated with collagen IV (Ibidi #8082) at a confluence of around 40%–50%. Next day, cells incubated with DMSO or with inhibitors were live imaged every 15 minutes for around 100–120 hours using Nikon A1 confocal microscope. The resulting time-lapse, Z-stacked images were spot-tracked manually using image analysis software IMARIS to identify precise locations of cell mitoses and various phases of the cell cycle.

### Lentivirus-Mediated ERK5 Silencing

MDA-MB-453 cells were stably transduced with shRNA lentiviral particles (Sigma-Aldrich #TRCN0000197264) to target the 3′ UTR of the *ERK5* transcript. shScr lentiviral particles (Sigma-Aldrich #SHC016V) were utilized as control. For viral infections, cells were incubated for 24 hours with viral particles at 1 to 2 MOI in the presence of 8 μg/mL polybrene. Infected cells were subsequently selected by incubation with 3 μg/mL puromycin until no live cells remained in the noninfected group (at least 3 days). Resistant colonies were pooled and expanded in puromycin-free–containing medium.

### Mammary Tumor Grafts

Female immunodeficient NSG mice purchased at 6 weeks old (Jackson Laboratory) were allowed to acclimatize in caged groups of five for 2 weeks before being injected. A total of 20 × 10^6^ MDA-MB-453 cells in 70 μL of Matrigel (BD Biosciences):PBS (1:1) were injected in the mammary fad pad (fourth nipple) on both sides. Once tumors had reached a palpable size [around 80 mm^3^ by caliper measurements using the formula = 0.5 × (length × width × depth)], mice were treated daily by oral gavage with 100 μg/kg lapatinib. Tumor size was subsequently determined twice weekly for 21 days, at which point mice were euthanized and each tumor was processed to obtain paraffin sections. Five animals per group were utilized for statistical analysis to confirm the effect and rule out artefacts associated with biological variability.

### Kaplan–Meier Analysis

Kaplan–Meier plots were generated through the Jetset best probes (dataset number # “35617_at” for *ERK5*, # “211370_s_at” for *MEK5*) to evaluate the prognostic significance of ERK5 and MEK5 in relapse-free survival (RFS) and distant metastasis-free survival (DMFS) (http://www.kmplot.com). Patient specimens were divided into high and low expression groups according to the median expression. The median was computed before any subgroups were analyzed. HRs with 95% confidence intervals and log-rank *P* values were calculated automatically by the website software.

### Statistical Analysis

Data are shown as means and SDs of the means. Statistical significance was calculated using a paired Student *t* test for direct comparison of two conditions and this was performed using the GraphPad Prism 7.0 software. The cutoff for statistical significance was set at *P ≤ 0.05.

### Data Availability Statement

The data generated in this study are available within the article and its Supplementary Data files.

## Results

### ERK5 Expression Is Associated with Increased Risk of Malignant Recurrence of *HER2*^+^ Breast Cancer

**FIGURE 1:**
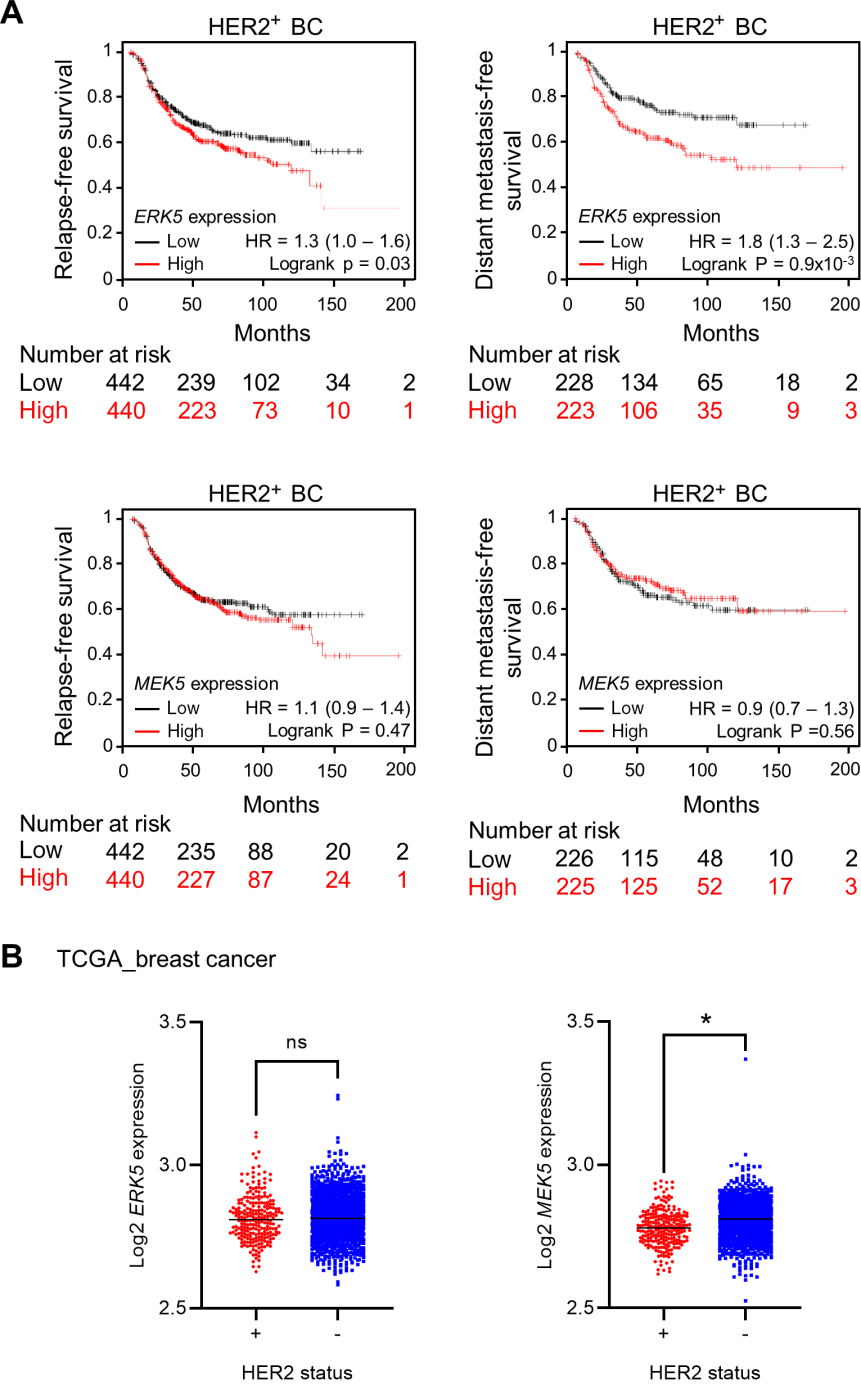
The ERK5 pathway strongly associates with poor prognosis in HER2^+^ breast cancer. **A,** The relationship between the level of expression of *ERK5* or *MEK5* and RFS or DMFS, of HER2^+^ breast cancer patients was assessed by Kaplan–Meier plots. Samples were divided into two groups with high (red) and low (black) expression. HRs and log-rank *P* values are shown. **B,** The level of *ERK5* and *MEK5* transcripts was analyzed from the breast cancer TCGA dataset of distinct molecular subtypes consisting of 1,668 HER2^−^ tumors and 236 HER2^+^ tumors. Black lines in each group indicate median with interquartile range. *P < 1 × 10^−4^ (Student unpaired *t* test).

We and others have previously found that the level of *ERK5* mRNA inversely correlated with DMFS across all breast cancers, in particular the basal-like molecular subtype (25–29). In this study, we explored the association of ERK5 signaling with RFS and DMFS in women diagnosed with HER2^+^ breast cancer using the Kaplan–Meier Plotter database tool (kmplot.com). We observed that high levels of *ERK5* in HER2^+^ tumors significantly correlated with early disease relapse and an increased risk of metastasis (*P* < 0.05, HRs: RFS = 1.3, and DMFS = 1.8; Fig. 1A). In contrast, high expression of *MEK5* mRNA was not a predictor of worse prognosis (*P* values > 0.05, HRs: RFS = 1.1 and DMFS = 0.9; Fig. 1A). Analyses of the transcriptomic breast cancer dataset acquired by The Cancer Genome Atlas (TCGA) Program (30) further showed that, whereas the level of the *MEK5* transcript was slightly higher, *ERK5* level was similar in HER2^−^ compared with HER2^+^ tumors, indicative of putative distinct mechanisms of ERK5 activation in different molecular subtypes of breast cancer (Fig. 1B). Unlike *ERK5*, no predictive outcome could be established from *ERK1/MAPK3* or *ERK2/MAPK1* expression levels (Supplementary Fig. S1).

**FIGURE 2:**
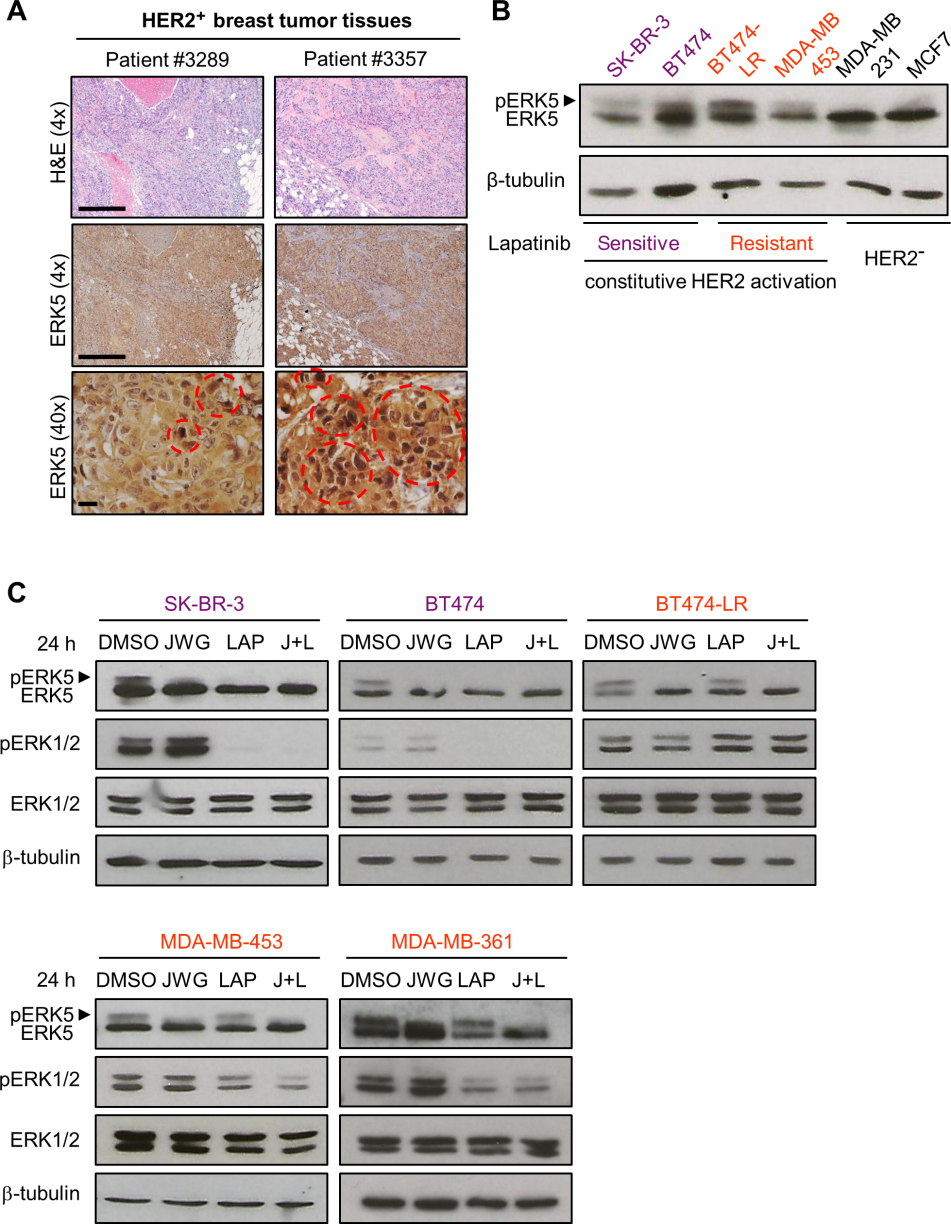
ERK5 is hyperphosphorylated across HER2-expressing breast cancer cell lines. **A,** Biopsies of breast tumor tissue from patients with HER2^+^ breast cancer were stained with H&E or with a specific antibody to ERK5 (brown). Scale bars: (4×) 500 μm, (40×) 20 μm. Areas of tumor exhibiting strong nuclear ERK5 staining are marked by red circles. **B** and **C,** Immunoblot analysis of ERK5 expression in human breast cancer cell lines. Where indicated (**C**), cells were mock treated with DMSO or incubated for 24 hours with 1 μmol/L lapatinib (LAP), 3 μmol/L JWG, or both (J+L). β-tubulin was utilized as a loading control. Similar results were obtained in three independent experiments.

To gain further insights into the clinical relevance of ERK5, we retrospectively analyzed the pattern of ERK5 expression in a cohort of patients diagnosed with invasive ductal HER2^+^ carcinoma. We selected 7 patients based on high expression of HER2 as determined by IHC. As previously reported (29), ERK5 was detected in normal glandular breast tissue and was present in both the nucleus and cytoplasm of mammary ductal and acinar cells as well as in the endothelial cells of blood vessels (Supplementary Fig. S2). In contrast, malignant HER2^+^ carcinoma tissue appeared highly disorganized with a number of cells of irregular size and shape containing a large nucleus with prominent ERK5 staining (Fig. 2A; Supplementary Fig. S2). The nuclear translocation of ERK5 in the tumor tissues was indicative of a conformational change caused by dual phosphorylation of ERK5 by MEK5 and increased ERK5 activity (22). Depending on the patient, the number of these cancer cells with robust nuclear ERK5 staining varied from a few (e.g., patient 3289) to many (e.g., patient 3357).

### Evidence for ERK5 as a Mediator of Lapatinib Resistance in HER2^+^ Breast Cancer Cells

We subsequently interrogated a panel of breast cancer cell lines featuring HER2 overexpression to decipher the role of ERK5 in HER2^+^ tumors (31). Specifically, these were (i) SK-BR-3 cells obtained from a malignant pleural effusion of breast adenocarcinoma that model a HER2^+^ subtype lacking estrogen and progesterone receptor expression (ER^−^/PR^−^), (ii) BT474 and BT474-derived lapatinib-resistant (BT474-LR) cell lines which represent HER2^+^ luminal (ER^+^/PR^+^) primary ductal carcinoma cells, and (iii) two further lapatinib-resistant breast cancer cell lines, namely MDA-MB-453 cells isolated from a malignant pericardial effusion of breast metaplastic (ER^−^/PR^−^) carcinoma exhibiting high *HER2* mRNA levels and MDA-MB-361 cells isolated from a brain metastatic site of HER2^+^ luminal (ER^+^/PR^−^) adenocarcinoma. According to their *HER2* gene amplification status, SK-BR-3, BT474, BT474-LR, and MDA-MB-361 cell lines exhibited higher levels of expression and phosphorylation of HER2 than MDA-MB-453 cells (Supplementary Fig. S3A). We further demonstrated that 1 μmol/L lapatinib treatment inhibited the phosphorylation of all tyrosine sites on HER2 in both sensitive and resistant breast cancer cells (Supplementary Fig. S3A and S3B). Consistent with its dual inhibitory effect on HER2 and HER1/EGFR/ERBB1 (32), lapatinib treatment blocked the phosphorylation of HER1 at Tyr1068 (Supplementary Fig. S3C). The phosphorylation of HER3/ERBB3 at Tyr1289 was also blocked by lapatinib as a downstream consequence of HER1/HER2 inhibition (Supplementary Fig. S3C). As expected (20), lapatinib suppressed AKT phosphorylation in both sensitive and resistant cell lines (Supplementary Fig. S4A).

We next determined the activation status of ERK5 in the HER2^+^ cell lines by monitoring its hyperphosphorylation via a characteristic mobility shift on SDS-polyacrylamide gels (24). ERK5 hyperphosphorylation was detected in all the HER2^+^ cell lines (Fig. 2B). Interestingly, the proportion of hyperphosphorylated ERK5 in BT474-LR cells was noticeably higher than that in the lapatinib-sensitive parental BT474 cell line. Conversely, no mobility shift was detected in triple-negative (ER^−^/PR^−^/HER2^−^) MDA-MB-231 or in HER2^−^ luminal (ER^+^/PR^+^) MCF-7 cells (Fig. 2B). We confirmed that the slow migrating form of ERK5 detected in HER2^+^ breast cancer cell lines disappeared following the suppression of ERK5 activity using the ERK5 inhibitor JWG-045 that exhibits significantly less affinity for BRD4 compared with XMD8–92 (refs. 33, 34; Fig. 2C). Inhibition of ERK5 hyperphosphorylation was also achieved by incubating the lapatinib-sensitive SK-BR-3 or BT474 cells with 1 μmol/L lapatinib (Fig. 2C). In contrast, lapatinib treatment did not significantly change the level of hyperphosphorylated ERK5 in the lapatinib-resistant BT474-LR, MDA-MB-453, or MDA-MB-361 cells (Fig. 2C). Similar to the pattern with ERK5, lapatinib blocked ERK1/2 phosphorylation in sensitive, but not in resistant cell lines, although partial inhibition of ERK1/2 phosphorylation was observed in lapatinib-treated MDA-MB-361 cells (Fig. 2C). Collectively, these results indicated that ERK5 and ERK1/2 were constitutively activated in lapatinib-resistant HER2^+^ breast cancer cells.

**FIGURE 3:**
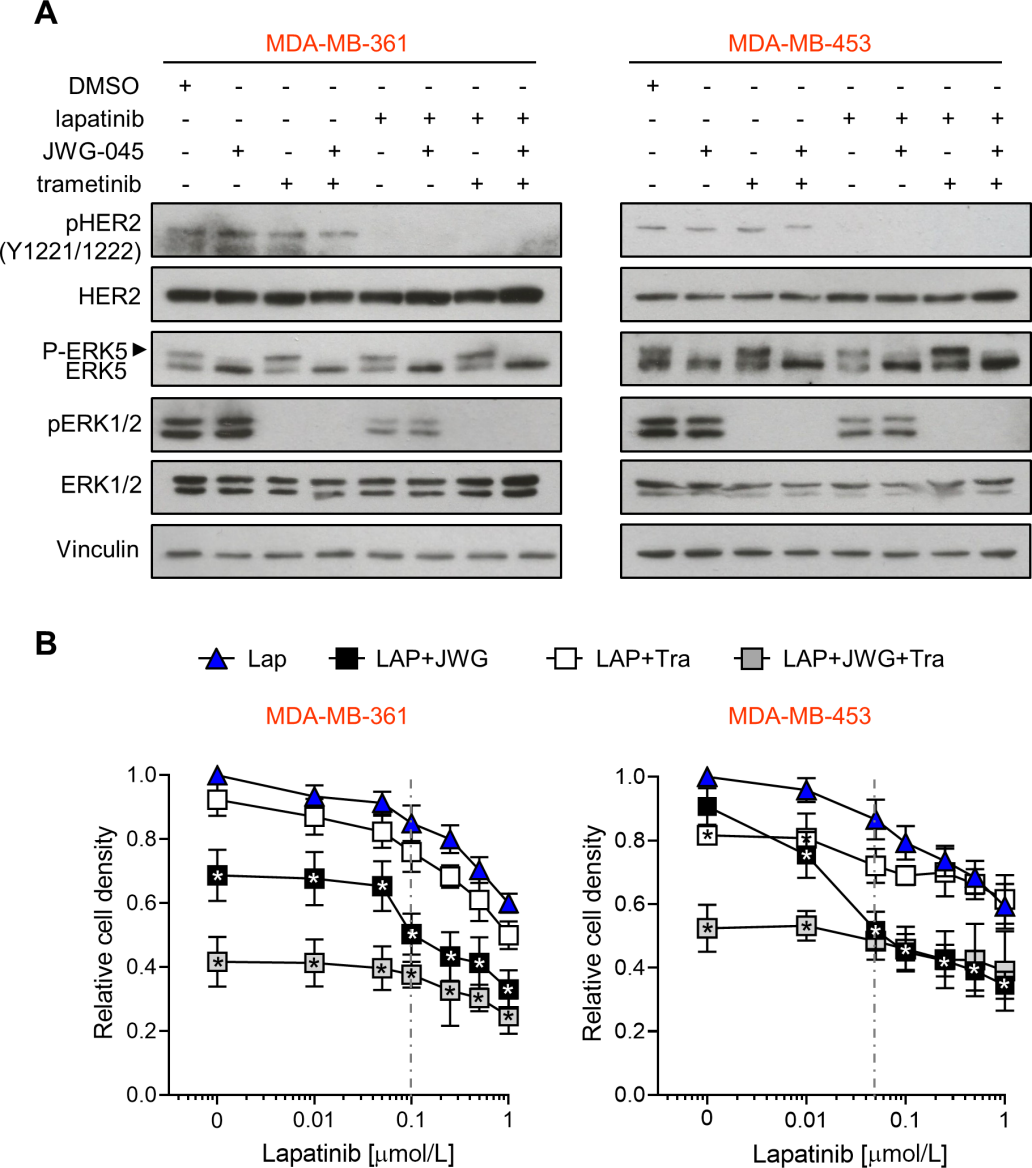
Inhibition of ERK5 enhances HER2^+^ breast cancer cell sensitivity to lapatinib. **A,** Subconfluent MDA-MB-361 and MDA-MB-453 cells were starved overnight in 0.1% FBS prior to being mock treated with DMSO or incubated with lapatinib (LAP; 500 nmol/L) alone or in combination with 3 μmol/L JWG-045 (LAP+JWG), 100 nmol/L trametinib (LAP+Tra), or 3 μmol/L JWG-045 plus 100 nmol/L trametinib (LAP+JWG+Tra) for 24 hours in FBS-containing media. Protein lysates were analyzed by immunoblot. Similar results were obtained in two independent experiments. **B,** Cells were cultured in 10% FBS-containing media with the indicated concentrations of LAP, with or without ERK5 (JWG; 3 μmol/L), and/or MEK1/2 (Tra; 100 nmol/L) inhibitors, for 9 days (MDA-MB-361) or 6 days (MDA-MB-453) prior to being fixed and stained with crystal violet. Cell density corresponds to the mean OD at 590 nm ± SD from three biological repeats performed in duplicate. The data are presented as fold of mock treated cells with DMSO. The results show that lapatinib plus JWG-045 exerts a stronger inhibitory effect on cell number compared with lapatinib alone or lapatinib plus trametinib; *P < 0.05 indicates statistical differences between cell densities of different treatment groups compared with the same concentration of lapatinib as a single agent.

Compensatory activation of ERK1/2 as a consequence of PI3K inhibition constitutes a potential mechanism implicated in resistance to anti-HER2 therapy (35, 36). Therefore, we sought to distinguish the requirement for sustained ERK5 activation versus ERK1/2 activation in counteracting the antitumor activity of lapatinib. This was addressed by comparing the response of growing MDA-MB-361 and MDA-MB-453 cells to increasing concentrations of lapatinib in combination with either 3 μmol/L JWG-045 or 100 nmol/L of trametinib (ref. 37; a clinically relevant inhibitor of MEK1/MAP2K1 and MEK2/MAP2K2, the upstream activators of ERK1/2). We confirmed that constitutive phosphorylation of ERK5 in resistant cell lines was unaffected by lapatinib treatment, while ERK1/2 was partially inhibited in MDA-MB-361 cells (Fig. 3A). Interestingly, inhibition of MEK1/2 by trametinib notably increased the proportion of hyperphosphorylated ERK5 (Fig. 3A). This effect was observed across all HER2^+^ breast cancer cell lines and was also observed in resistant cells treated with PD0325901, another specific inhibitor of MEK1/2 (Supplementary Fig. S4B). In contrast, we found no evidence for a compensatory increase in ERK1/2 phosphorylation following ERK5 inhibition by JWG-045 (Figs. 2C and 3A).

Interestingly, MDA-MB-361 and MDA-MB-453 cells exhibited distinct sensitivity to JWG-045 and trametinib as single agents, with MDA-MB-361 cells being markedly more sensitive to ERK5 inhibition than MDA-MB-453 cells (Fig. 3B; [LAP] = 0). Nonetheless, dual inhibition of ERK1/2 and ERK5 severely impaired the growth of both cell types to a similar extent. But most importantly, JWG-045 was significantly more effective than trametinib at decreasing cell densities across the dose response of lapatinib concentrations (Fig. 3B). In addition, from around 50 or 100 nmol/L to 1 μmol/L lapatinib, trametinib did not further enhance the growth-inhibitory effect of JWG-045 in combination therapy (Fig. 3B). This observation could suggest that the level of inhibition of AKT activity by lapatinib might constitute a rate-limiting factor for allowing ERK1/2 to compensate for the loss of ERK5. Collectively, these data strongly implied that sustained hyperphosphorylation of ERK5 played a relevant role in resistance to HER2 inhibition. Moreover, the ability of ERK1/2 to compensate for the loss of ERK5 appeared to be dependent on the level of AKT activity.

### ERK5 Confers Resistance to the Antiproliferative Effect of Lapatinib

**FIGURE 4:**
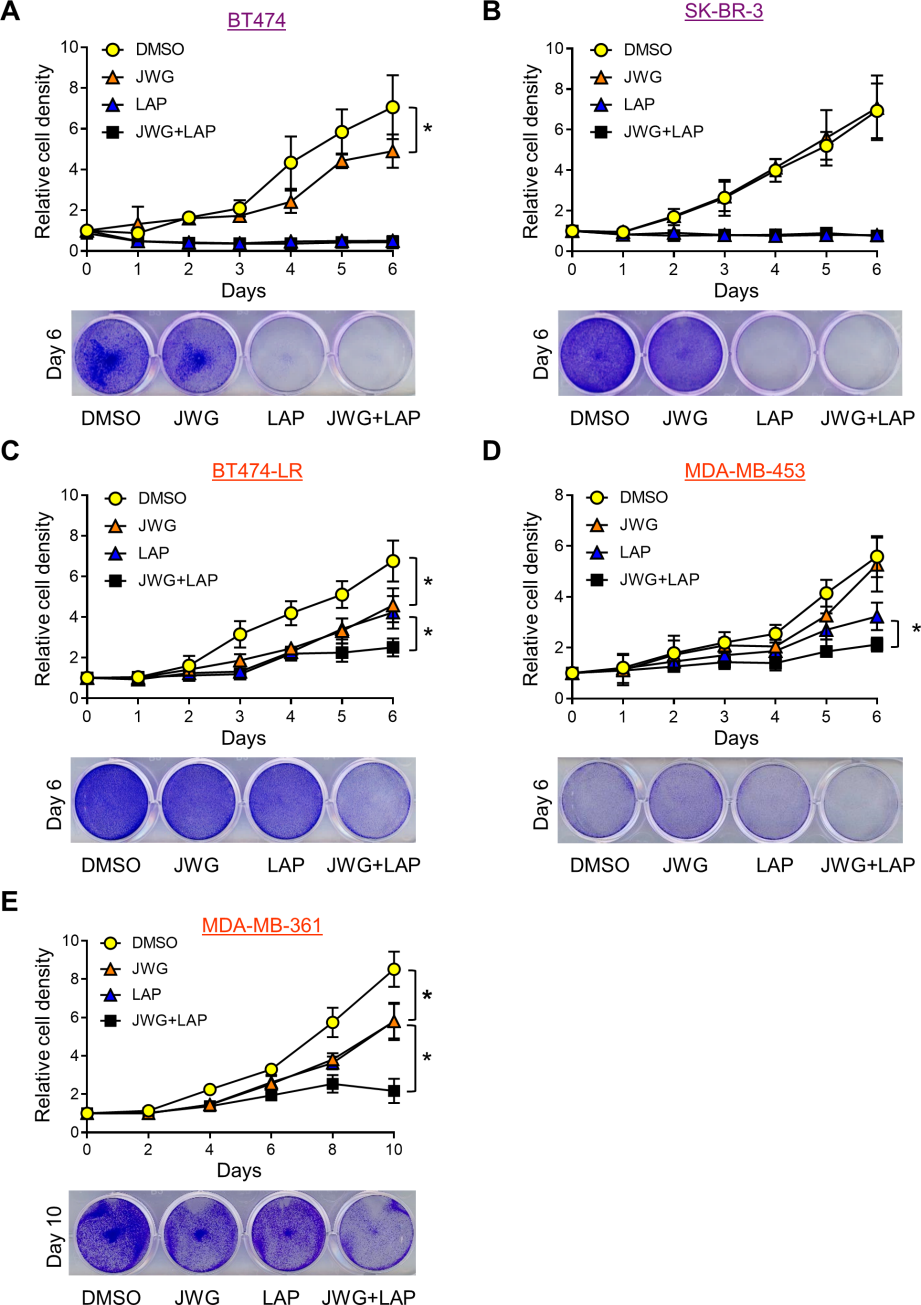
Constitutive ERK5 activation promotes lapatinib resistance. BT474 (**A**), SK-BR-3 cells (**B**), BT474-LR (**C**), MDA-MB-453 (**D**), and MDA-MB-361 (**E**) cell lines were cultured for several days in media supplemented with 10% FBS and containing DMSO (mock treated), 3 μmol/L JWG-045 (JWG), 500 nmol/L lapatinib (LAP), or a combination of 500 nmol/L lapatinib plus 3 μmol/L JWG-045 (JWG+LAP). Cell density was estimated by crystal violet staining. Mean OD at 590 nm ±SD of three biological repeats performed in duplicate are presented as fold relative to DMSO-treated controls. *P < 0.05 indicates statistical differences between cell densities of different treatment group at the end of the experiment (day 6 or day 10). Representative photos of crystal violet staining of breast cancer cells at the end of the experiment (day 6 or day 10). show that combined HER2–ERK5 inhibition significantly reduced the number of BT474-LR, MDA-MB-453, and MDA-MB-361 cells compared with single-agent treatment.

To validate the benefit of anti-ERK5 therapy in the context of treatment-resistant HER2^+^ breast cancers, we performed detailed comparative analyses of breast cancer cell sensitivity to JWG-045, lapatinib or combined treatment. For these experiments, we selected a concentration of 500 nmol/L lapatinib that achieved a marked cytotoxic effect in sensitive, but not in resistant, cell lines (Supplementary Fig. S5). Accordingly, 500 nmol/L lapatinib suppressed the growth of BT474 and SK-BR-3 cells (Fig. 4A and B), whereas only partially decreasing by around 40%/45% BT474-LR, MDA-MB-453, and MDA-MB-361 cell densities (Fig. 4C–E). Moreover, we found that HER2^+^ breast cancer cell lines displayed distinct sensitivity to ERK5 inhibition, independently of their resistance status to lapatinib. Specifically, sole treatment with JWG-045 had no impact on SK-BR-3 and MDA-MB-453 cell lines (Fig. 4B and D), but reduced BT474 and BT474-LR cell numbers by around 30%/35% (Fig. 4A and C) and that of MDA-MB-361 cells by 25% (Fig. 4E). Regardless of the differential effect of JWG-045 as a single treatment, ERK5 inhibition significantly enhanced the sensitivity of all the resistant cell lines to lapatinib (Fig. 4C–E). This did not correlate with increased apoptotic cell death (Supplementary Fig. S5). Consequently, we assessed the activity of JWG-045 alone and in combination treatment on cell-cycle progression by flow cytometry.

**FIGURE 5:**
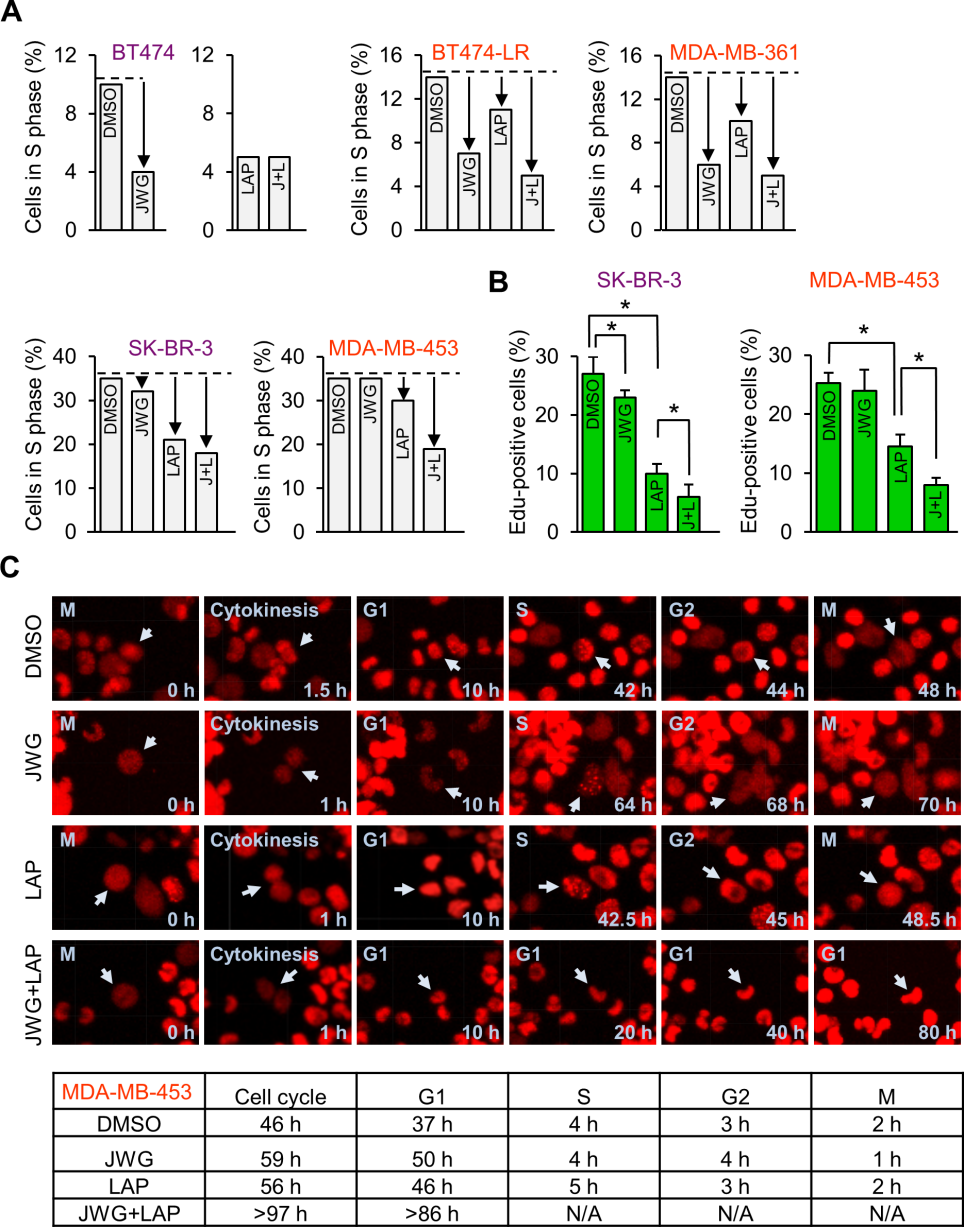
Combined HER2–ERK5 inhibition causes G_1_ cell-cycle arrest. **A** and **B,** HER2^+^ breast cancer cell lines starved overnight in 0.1% FBS were stimulated for 24 hours with 10% FBS in media containing DMSO, JWG-045 (JWG; 3 μmol/L), lapatinib (LAP; 500 nmol/L), or a combination of 3 μmol/L JWG-045 plus 500 nmol/L lapatinib (JWG+LAP). Quantification of percentages of cells in S-phase identified by flow cytometry after PI staining of duplicate samples are presented (**A**). The data obtained for BT474 cells are presented in two separate graphs to indicate that different voltages were employed for analyzing the intensity of PI staining. Similar patterns were obtained in three independent experiments performed in duplicate. Alternatively, SK-BR-3 and MDA-MB-453 cells were analyzed by immunofluorescence imaging of EdU staining. The graphs show the quantification of mean percentages of Edu-positive cells ± SD from three independent experiments (**B**). At least 200 cells were counted per treatment. *P < 0.05. **C,** MDA-MB-453 cells transduced with an mCherry-PCNA reporter were analyzed by single-cell live imaging. Example images of single MDA-MB-453 cells over time expressing mCherry-PCNA are shown. While growth phases of the cell cycle (G_1_ and G_2_) exhibit a smooth nuclear PCNA staining, S-phase is identified by punctate PCNA staining marking DNA replication foci. The PCNA protein spills over the entire cell during mitosis due to the breakdown of the nuclear envelope. The mean period of time cells spend in each phase of the cell cycle between mitosis is indicated in the table (*N* ≥ 20 cells).

The exquisite sensitivity of BT474 cells to 500 nmol/L lapatinib treatment was demonstrated by a marked increase in the number of cells in sub-G_1_, which coincided with a significant loss in viable cells after 24-hour incubation (Supplementary Fig. S6). To account for the reduction in BT474 cell numbers, flow cytometry voltage settings were adjusted to generate G_1_ peaks in the same area on the histograms. Consequently, the percentages of lapatinib-treated BT474 cells in S phase are presented in a separated graph because these data are not directly comparable with that acquired for DMSO- and JWG-045–treated samples (Fig. 5A). Unlike BT474 cells, lapatinib reduced the percentage of SK-BR-3 cells in S-phase without decreasing cell viability after 24 hours and exhibited a limited impact on BT474-LR, MDA-MB-453, and MDA-MB-361 cell-cycle profiles (Fig. 5A; Supplementary Fig S6). Moreover, JWG-045 exerted a strong antiproliferative activity in BT474, BT474-LR, and MDA-MB-361, but did not affect the proliferation of SK-BR-3 and MDA-MB-453 cells. Importantly, the greatest degree of cell-cycle arrest was achieved upon combination treatment (Fig. 5A; Supplementary Fig S6). This was particularly significant in the resistant MDA-MB-453 cell line given their limited sensitivity to single-agent activity. MDA-MB-361 and MDA-MB-453 cells arrested in G_1_ for several days appeared to regrow after the removal of the drugs, indicating that JWG-045 enhanced the cytostatic activity of lapatinib in resistance cells without increasing cytotoxicity (Supplementary Fig. S7A).

The impact of ERK5 inhibition in the context of HER2-targeted therapy was further assessed by Edu labeling of proliferating breast cancer cells. As expected, lapatinib alone exerted a stronger effect in SK-BR-3 cells compared with MDA-MB-453 cells, as indicated by 70% versus 40% reduction in the number of Edu^+^ cells, respectively (Fig. 5B; Supplementary Fig. S7B). Conversely, JWG-045 did not markedly affect Edu staining of SK-BR-3 and MDA-MB-453 cells consistent with the lack of requirement of ERK5 for mediating SK-BR-3 and MDA-MB-453 cell-cycle progression through G_1_–S. Nonetheless and in line with our previous findings, combination treatment resulted in the most notable reduction in the number of dividing cells (Fig. 5B). We further compared the effect of single agent versus combination treatment on cell-cycle kinetics of MDA-MB-453 cells using a single-cell live imaging approach (38). Specifically, MDA-MB-453 cells were transduced with a lentiviral vector expressing an mCherry-PCNA fusion construct to allow the phases of the cell cycle to be distinguished (Fig. 5C). In growing conditions on collagen IV–coated plates, the majority of MDA-MB-453 cells completed a full cell cycle in under 50 hours (Fig. 5C). Incubation with JWG-045 or lapatinib alone slowed this down to around 60 hours as a result of an increased period of time the cells spent in G_1_. Remarkably, no cells were able to transition from G_1_ to S-phase after treatment with JWG-045 plus lapatinib (Fig. 5C), thereby providing additional evidence that combination treatment successfully overcame lapatinib resistance by causing a robust G_1_ cell-cycle arrest.

### ERK5 Inhibition Achieves Therapeutic Sensitivity Through Suppressing RB Phosphorylation

**FIGURE 6:**
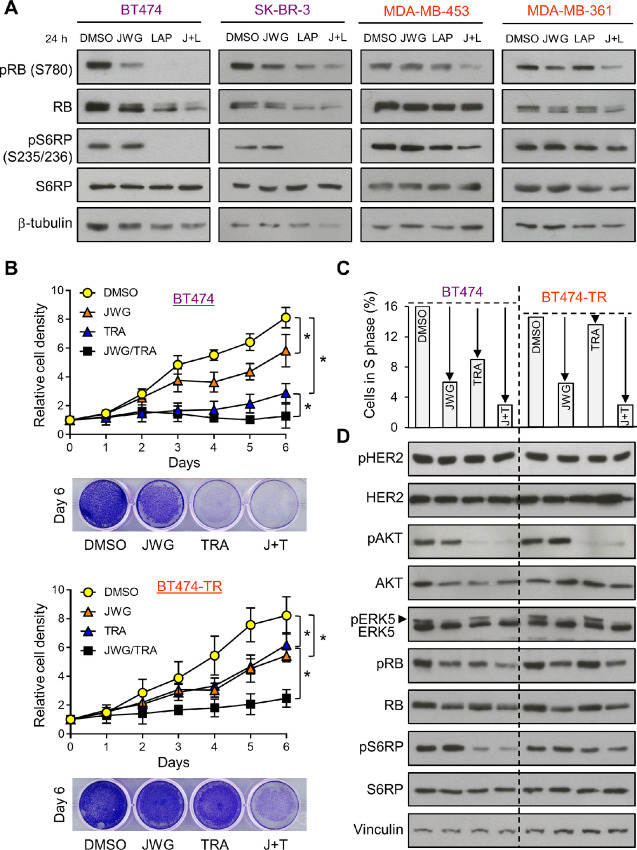
ERK5 inhibition enhances anti-HER2 therapeutic efficacy by reducing RB phosphorylation. **A,** HER2^+^ breast cancer cell lines starved overnight in 0.1% FBS were stimulated for 24 hours with 10% FBS in media containing DMSO, JWG-045 (JWG; 3 μmol/L), lapatinib (LAP; 500 nmol/L), or a combination of 3 μmol/L JWG-045 plus 500 nmol/L lapatinib (J**+**L). Protein lysates were analyzed by immunoblot with the indicated antibodies. β-tubulin expression was used as loading control. Similar results were obtained in two independent experiments. **B**–**D,** BT474 and BT474-TR cells starved overnight in 0.1% FBS were stimulated with 10% FBS in media containing DMSO, JWG-045 (JWG; 3 μmol/L), trastuzumab (TRA; 10 μg/mL), or a combination of 3 μmol/L JWG-045 plus 10 μg/mL trastuzumab (J+T). Adherent cells were stained by crystal violet and cell density presented as fold relative to DMSO-treated controls corresponds to the mean OD at 590 nm ±SD of three biological repeats performed in duplicate (**B**). *P < 0.05 indicates statistical differences between cell densities of different treatment group at day 6. Representative photos of crystal violet staining of breast cancer cells at day 6 show that combined treatment with JWG-045 plus trastuzumab further reduced the number of BT474-TR cells compared with each treatment alone. Alternatively, cells were incubated with inhibitors for 24 hours and labeled with PI to quantify the percentages of cells in S phase by flow cytometry (**C**) or harvested for protein analyses by immunoblot with the indicated antibodies (**D**). β-tubulin expression was used as loading control. Similar results were obtained in three independent experiments.

Having found that ERK5 inhibition increased the antiproliferative effect of lapatinib, we next investigated the molecular interaction between ERK5 and HER2 signaling in breast cancer cells. As expected, lapatinib as a single treatment inhibited the phosphorylation of RB (a key mediator of G_1_–S transition; ref. 39), and S6RP/RPS6 (a readout of S6K activity involved in G_1_ progression downstream of the PI3K/AKT/mTOR pathway; ref. 40) in sensitive cell lines (Fig. 6A). RB phosphorylation was also suppressed by JWG-045 to a degree that matched its distinct capability to cause a G_1_ arrest in different HER2^+^ breast cancer cell lines (Fig. 6A). In particular, in conditions where lapatinib had no impact, JWG-045 notably reduced RB phosphorylation in the resistant MDA-MB-361 cell line. Consistent with this, we had observed that MDA-MB-361 cells were more sensitive to the antiproliferative activity of JWG-045 than that of lapatinib treatment after 24-hour incubation (Fig. 5A). The requirement of ERK5 for mediating RB phosphorylation was further supported by evidence that incubation of MDA-MB-361 cells with a different inhibitor of ERK5, namely AX-15836 (41), or with the MEK5 inhibitor BIX-02189 (42) caused a reduction in the level of phospho-RB (Supplementary Fig. S8A). The suppressive effect of ERK5 signaling blockade on RB coincided with decreased cell growth, as demonstrated by reduced densities of MDA-MB-361 cells incubated with AX-15836 or BIX-02189, alone or in combination therapy (Supplementary Fig. S8B). Combination treatment also caused a marked reduction in the level of RB phosphorylation in MDA-MB-453 cells where single-agent treatment had no effect (Fig. 6A).

Next, we sought to further establish the significance of ERK5-mediated RB phosphorylation in breast cancer cell resistance following HER2 pathway blockade. This was achieved by conducting a comparative analysis of mAb therapy in parent BT474 cells and a derivative trastuzumab-resistant cell line (BT474-TR) generated by prolonged culture in trastuzumab. As expected, BT474-TR cell density was less impacted by trastuzumab than that of BT474 cells (Fig. 6B) with the agent exerting more potent apoptotic and antiproliferative activities in BT474 than in BT474-TR cells (Fig. 6C; Supplementary Fig. S9A and S9B). However, BT474-TR cells remained sensitive to ERK5 inhibition by JWG-045, AX-15836 or BIX-02189 (Fig. 6B; Supplementary Fig. S10A). The antiproliferative effect of JWG-045 in BT474 cells was noticeably stronger than that of trastuzumab (Fig. 6C). At the molecular level, trastuzumab inhibited AKT phosphorylation in both sensitive and resistant cell lines, without affecting the phosphorylation status of HER2 or ERK5 (Fig. 6D). In addition, trastuzumab reduced the level of RB and S6RP phosphorylation in BT474 cells but not in BT474-TR cells (Fig. 6D). Conversely, JWG-045 decreased RB phosphorylation in both cell lines (Fig. 6A–D). A similar reduction in the level of phospho-RB was reproduced in BT474-TR cells incubated with AX-15836 or BIX-02189 (Supplementary Fig. S10B). Importantly, JWG-045 restored the growth-inhibitory effect of trastuzumab in BT474-TR cells to the level achieved by trastuzumab monotherapy in BT474 cells (Fig. 6B). Increased therapeutic sensitivity of both sensitive and resistant BT474 cell lines to trastuzumab following ERK5 inhibition coincided with the strongest reductions in the proportion of cells in S-phase (Fig. 6C). Collectively, these observations strengthened our evidence that ERK5 inhibition enhanced the antiproliferative activity of HER2 inhibitors in resistant breast cancer cells.

### ERK5 Silencing Restores Lapatinib Sensitivity of Resistant Tumor Grafts *In Vivo*

To demonstrate the relevance of ERK5 in resistance to anti-HER2 therapy, we performed an *in vivo* experiment utilizing MDA-MB-453 cells in which ERK5 expression was silenced by stable expression of an shRNA targeting the 3′UTR of the *ERK5* transcript. Consistent with our previous data, the loss of ERK5 signaling alone did not affect MDA-MB-453 cell growth, but significantly enhanced MDA-MB-453 cell sensitivity to lapatinib without affecting the level of phosphorylation of ERK1/2 (Supplementary Fig. S11A and S11B). Accordingly, combined ERK5 silencing plus lapatinib treatment caused a greater reduction in the percentage of breast cancer cells in S-phase compared with lapatinib alone (Supplementary Fig. S11C).

**FIGURE 7:**
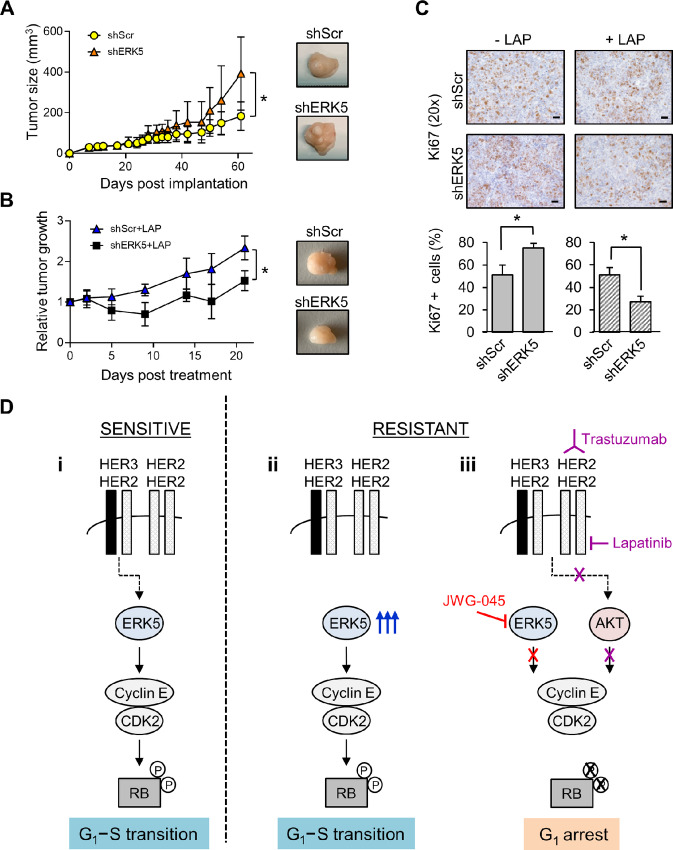
ERK5 silencing enhanced the anti-tumor activity of lapatinib in a resistant mammary graft model. MDA-MB-453 cells carrying shScr or shERK5 were orthotopically transplanted into the mammary fad pad of NSG mice. In **B**, animals carrying small tumors received lapatinib by oral gavage. **A** and **B,** Tumor volumes were measured twice a week for the duration of the experiment. The data presented as tumor size (**A**) or fold increase in tumor size (**B**) correspond to the mean ± SD (*n* = 5 mice in each group). Representative pictures of tumor grafts excised from mice sacrificed at the end of the experiment are shown. **C,** Sections of mammary graft tumors were analyzed by immunohistochemistry using a specific antibody to Ki67. Scale bar, 50 μm. **D,** Model of RB and S6RP phosphorylation in resistant breast cancer cells. (i) In breast cancer cells, ERK5-mediated RB phosphorylation by cyclin E-CDK2 downstream of HER2/HER3-heterodimers promotes G_1_–S transition. (ii) In resistant cells, constitutive activation of ERK5 independently of HER2/HER3 (e.g., through an IGF1R/SRC axis) renders these cells less sensitive to HER2-targeted therapy. (iii) When JWG-045–treated resistant cells are cotreated with anti-HER2 therapy (e.g., lapatinib, trastuzumab) to inhibit AKT downstream of HER2 dimers, maximal reduction in RB is observed trough complete inhibition of the cyclin E-CDK2 complex, causing a G_1_ arrest.

The MDA-MB-453 cells were subsequently transplanted into the mammary fat pad of athymic female NSG mice to allow orthotopic tumor formation. Once tumors were detectable, randomized cohorts of mice carrying size-matched control and ERK5 shRNA mammary grafts on each flank were continuously treated with lapatinib for the duration of the experiment. Significant differences in MDA-MD-453 tumor growth were observed between the different cohorts. Specifically, although ERK5 downregulation on its own appeared to accelerate tumor growth (Fig. 7A), the silencing of ERK5 significantly enhanced the antitumor activity of lapatinib, thereby confirming the relevant role of ERK5 signaling in mediating therapeutic resistance to anti-HER2 targeting (Fig. 7B). When tumors achieved the maximum tolerated volume, mice carrying orthotopic mammary xenografts were euthanized. Immunostaining of harvested tumor sections with an antibody to the proliferation marker Ki67 showed that tumor cell proliferation was increased in the absence of ERK5 (Fig. 7C). Nonetheless, ERK5 deficiency significantly reduced the percentage of Ki67^+^ cells in tumors exposed to lapatinib therapy (Fig. 7C). This observation adds further support to the idea that pharmacologic inhibition of ERK5 might constitute a potentially effective therapeutic strategy in the context of breast cancer resistant to anti-HER2–targeted agents.

## Discussion

Overexpression of the ERK5 MAPK in breast cancer results in poor survival (25–29). In this study, we found that sustained ERK5 hyperphosphorylation correlated with decreased sensitivity of HER2^+^ breast cancer cells to anti-HER2 agents. Collectively, our data demonstrated that inhibiting ERK5 overcame resistance to lapatinib or trastuzumab by causing a G_1_ cell-cycle arrest which correlated with a substantial decrease in the level of RB phosphorylation. Therapeutic resistance of HER2^+^ breast cancer has been linked to cyclin D1-CDK4/6-mediated RB phosphorylation (18–20) and also the overexpression of cyclin E and increased CDK2 activity (43). We previously demonstrated that ERK5 deficiency in mouse embryonic fibroblasts caused a growth arrest in the late-G_1_ phase by suppressing cyclin E-CDK2 activity, rather than cyclin D1–CDK4 activity, as a consequence of increased expression of p21/CDKN1A and p27/CDKN1B (44). In addition, mass spectrometry–based quantitative proteomics analysis indicated that CDK2 interacted with ERK5 in HeLa cells (45). It is therefore tempting to speculate that sustained ERK5 signaling neutralizes the antiproliferative effect of anti-HER2 agents in resistant breast cancer cells by promoting RB phosphorylation through the cyclin E–associated CDK2 complex (Fig. 7D).

The strongest antiproliferative effect of JWG-045 as a single treatment was observed in HER2^+^ breast cancer cell lines that were also hormone receptor–positive (ER^+^/PR^+/−^). In line with conflicting findings regarding the requirement of ERK5 in cell proliferation, several potential mechanisms can explain the distinct phenotypic outcome produced by ERK5 inhibition on RB phosphorylation in ER^−^/PR^−^/HER2^+^ breast cancer cells. For example, the limited effect of JWG-045 in SK-BR-3 and MDA-MB-453 cells might be a consequence of compensatory activation of cyclin E–CDK2 by AKT downstream of HER2 (43). Therefore, dual inhibition of ERK5 and AKT is required to effectively suppress cyclin E-CDK2–mediated phosphorylation of RB (Fig. 7D). Nonetheless, in spite of exhibiting no reduction in RB phosphorylation, MDA-MB-453 cells treated with JWG-045 as a single agent spent more time in G_1_ (Fig. 5C). Increased duration of the G_1_ phase was similar to that caused following lapatinib treatment. However, unlike lapatinib, JWG-045 did not impair cell density (Fig. 4D) nor did it affect the number of MDA-MB-453 cells in S-phase (Fig. 5A). Coating the coverslips with collagen IV for the live-imaging experiments might explain this apparent discrepancy. Components of the extracellular matrix (ECM) such as collagens have been shown to enhance the resistance of MDA-MB-453 cells to combined inhibition of HER2 and PI3K (46). Our evidence that MDA-MB-453 cells cultured on collagen IV were more sensitive to the antiproliferative effect of JWG-045 might suggest that inhibition of ERK5 in combination therapy could constitute a particularly important strategy for counteracting HER2^+^ breast tumor resistance acquired through elevated expression of ECM and cell adhesion genes. This idea is prompting future experiments to understand how enhanced ECM/integrin signaling would influence the requirement of ERK5 to phosphorylate RB.

Several receptor and nonreceptor tyrosine kinases could mediate sustained ERK5 activation in resistant HER2^+^ cell lines treated with lapatinib (47). Of particular relevance is the stimulation of the IGF1 receptor (IGF1R) which was found to form a heterotrimeric complex with HER2 and HER3 resulting in SRC activation in breast cancer cells (48–50). Consistent with the idea that the ERK5 pathway is stimulated downstream of HER2–HER3 complexes (24), trastuzumab, which acts by selectively blocking HER2 dimers (51), did not inhibit ERK5 hyperphosphorylation in BT474 breast cancer cells (Fig. 6C). Moreover, SRC and IGF1R were recently implicated in driving pancreatic ductal adenocarcinoma and melanoma resistance to pharmacologically or genetically targeted inhibition of ERK1/2 signaling through activating ERK5 (52, 53). Inhibition of MEK1/2 in colorectal cancer cells might also activate ERK5 by suppressing dual specificity phosphatases (DUSP; ref. 54). In agreement with these findings, we discovered that MEK1/2 inhibition enhanced the level of ERK5 hyperphosphorylation in HER2^+^ breast cancer cells. Moreover, dual inhibition of ERK1/2 and ERK5 signaling severely impaired the growth of HER2^+^ breast cancer cells. Therefore, although inhibition of ERK1/2 signaling alone did not significantly enhance the sensitivity of HER2^+^ lapatinib-resistant cells to lapatinib treatment, we predict that combined inhibition of MEK1/2 and ERK5 would constitute an important alternative strategy for the treatment of resistant tumors to HER2 blockade.

Consistent with our *in vitro* data that combined treatment with JWG-045 plus lapatinib impaired G_1_ to S-phase transition, we demonstrated that ERK5 silencing enhanced the antitumor activity of lapatinib in MDA-MB-453 tumors *in vivo* (Fig. 7B). Given that ERK5-deficient tumors treated with lapatinib exhibited reduced cell proliferation, we anticipate that, similar to ERK5 inhibition, ERK5 downregulation in the context of HER1/HER2 targeting causes a G_1_ cell-cycle arrest. On the other hand, ERK5 deficiency on its own accelerated the rate of tumor growth in spite of evidence that long-term ERK5 silencing did not give MDA-MB-453 cells a proliferative advantage *in vitro* (Fig. 7A; Supplementary Fig. S10B). We previously observed a similar phenomenon by depleting ERK5 in MDA-MB-231 tumors (29). Likewise, *KRAS*-mutant patient-derived xenograft tumors grew substantially faster in animals exposed to a different ERK5 inhibitor, XMD8–92 (52). This apparent paradox highlights the importance of tumor growth analyses *in vivo* for preclinical validations of drug targeting strategies. The unique structural and functional properties of ERK5 should also be considered to understand the correlation between the level of ERK5 expression and decreased disease-free survival (25–29). In particular, compared with ERK5 inhibition or ERK5 silencing, overexpression of ERK5 might cause distinct phenotypic abnormalities as a consequence of abnormal transcriptional activity through its C-terminal tail (55, 56). Therefore, future studies to analyze the impact of varying the amount of ERK5 protein on tumor growth will be critical to firmly establish the biological significance of overexpression of ERK5 in human breast cancer. Ultimately, the translational implication of our findings will necessitate pharmacological approaches *in vivo* based on testing specific inhibitors of ERK5 signaling, for example, BIX02189 and JWG-071 (57), in the context of HER2-targeted therapies. Opening this new window of therapeutic opportunities has great potential to overcome early and advanced HER2^+^ breast cancer resistance to small molecular inhibitors of HER2, as well as to improve the efficacy of mAbs in first-line treatment.

## Supplementary Material

Figure S1 Kaplan-Meier analyses of human HER2+ breast cancer samples show that no predictive outcome can be established from ERK1 or ERK2 expression levels.

Figure S2 The pattern of ERK5 expression was analyzed in normal breast and tumor tissues from a cohort of patients diagnosed with invasive ductal HER2+ carcinoma.

Figure S3 Lapatinib treatment inhibits tyrosine phosphorylation of HER1/2/3 in both sensitive and resistant breast cancer cells.

Figure S4 Both, sensitive and resistant HER2+ breast cancer cell lines exhibit impaired AKT phosphorylation following lapatinib treatment and increased ERK5 hyperphosphorylation following incubation with trametinib or PD0325901.

Figure S5 BT474, SK-BR-3, BT474-LR and MDA-MB-453 cell lines display distinct sensitivity to the apoptotic effect of 500 nM lapatinib.

Figure S6 The effect of lapatinib and JWG-045 as single treatments was compared to that achieved in combination therapy on the cell cycle profile of sensitive and resistant breast cancer cell lines.

Figure S7 These data support the conclusion that ERK5 inhibtion enhances the cytostatic activity of lapatinib in resistance cells without increasing cytotoxicity.

Figure S8 AX-15836 and BIX-02189 reproduce the inhibitory effect of JWG-045 on the level of phospho-RB and MDA-MB-361 cell density.

Figure S9 JWG-045 enhances the sensitivity of HER2+ breast cancer cells to trastuzumab through decreasing the proportion of cells in S phase, rather than increasing apoptotic cell death.

Figure S10 Inhibition of ERK5 by AX-15836 or MEK5 by BIX-02189 enhance HER2+ breast cancer cell sensitivity to trastuzumab through decreasing RB phosphorylation.

Figure S11 Combined ERK5 silencing plus lapatinib treatment caused a greater reduction in the percentage of breast cancer cells in S phase compared with lapatinib alone.
