# Supplementary figures and images for: Inhibiting ERK5 Overcomes Breast Cancer Resistance to Anti-HER2 Therapy By Targeting the G1–S Cell-Cycle Transition
Source: Cancer Res Commun. 2022 Mar 10;2(3):131–45. doi: 10.1158/2767-9764.CRC-21-0089 (PMC7613885; doi:10.1158/2767-9764.CRC-21-0089)

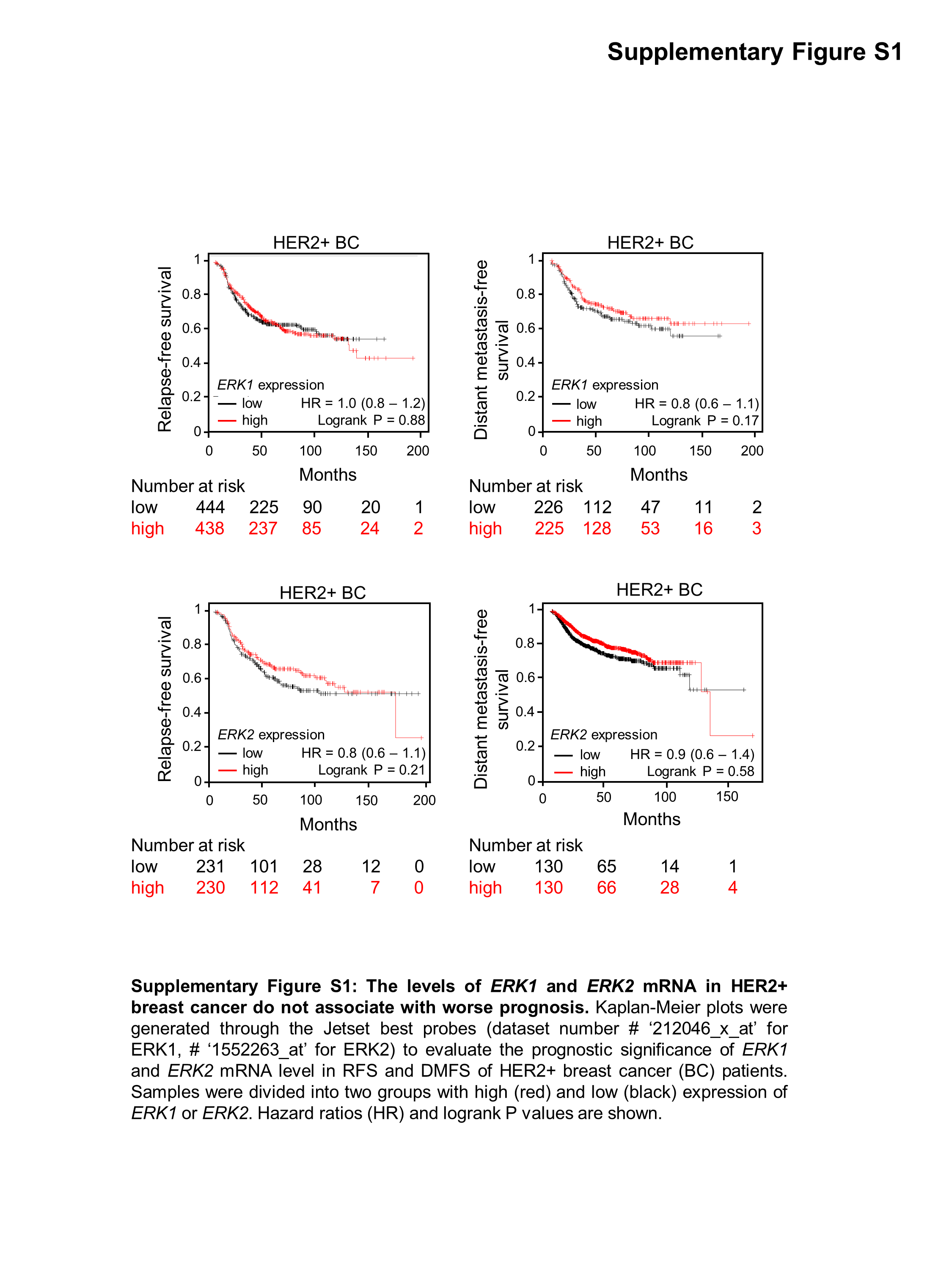

Supplement: Figure S1 — Kaplan-Meier analyses of human HER2+ breast cancer samples show that no predictive outcome can be established from ERK1 or ERK2 expression levels. [file crc-21-0089-s01.png]

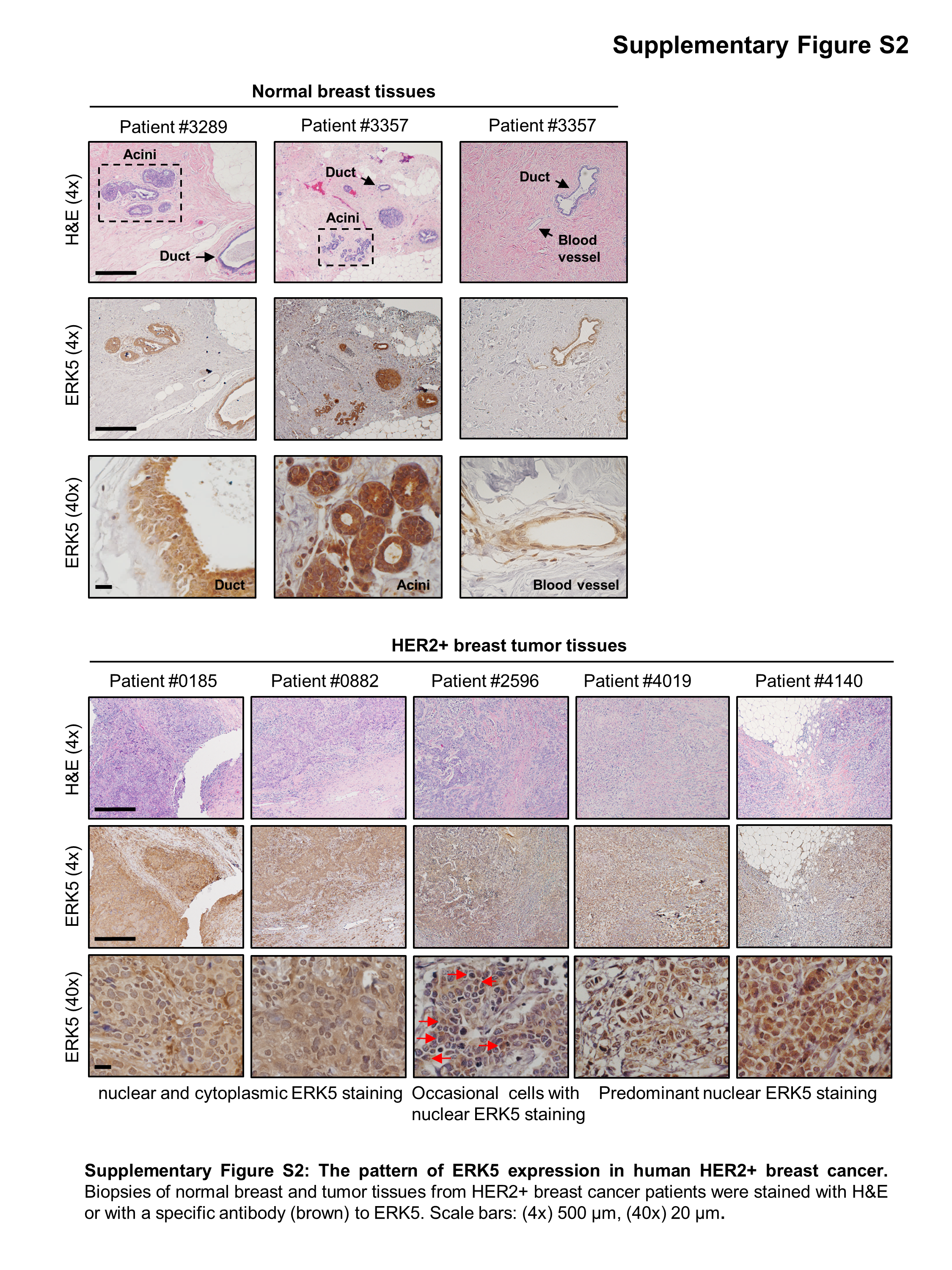

Supplement: Figure S2 — The pattern of ERK5 expression was analyzed in normal breast and tumor tissues from a cohort of patients diagnosed with invasive ductal HER2+ carcinoma. [file crc-21-0089-s02.png]

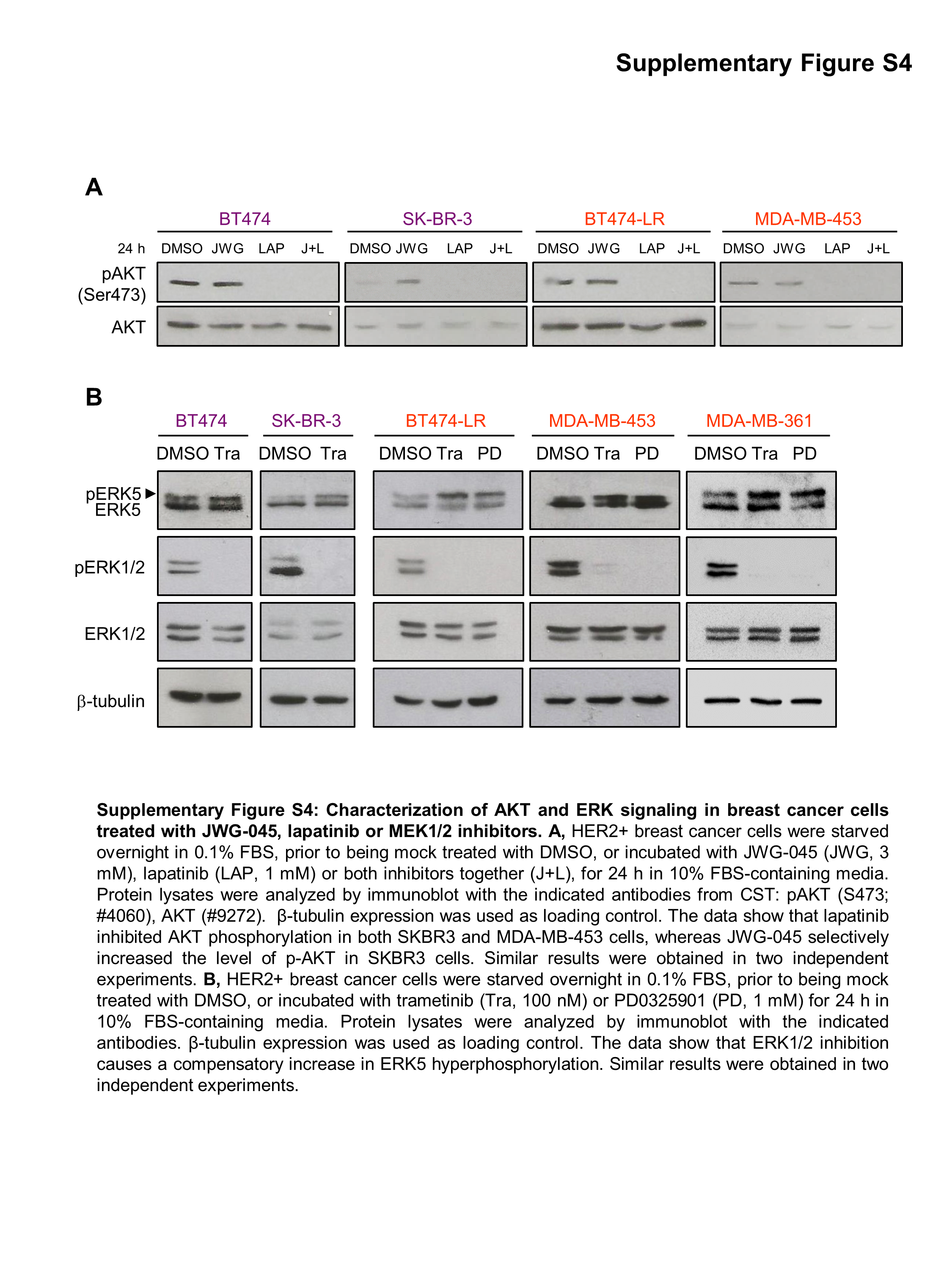

Supplement: Figure S4 — Both, sensitive and resistant HER2+ breast cancer cell lines exhibit impaired AKT phosphorylation following lapatinib treatment and increased ERK5 hyperphosphorylation following incubation with trametinib or PD0325901. [file crc-21-0089-s04.png]

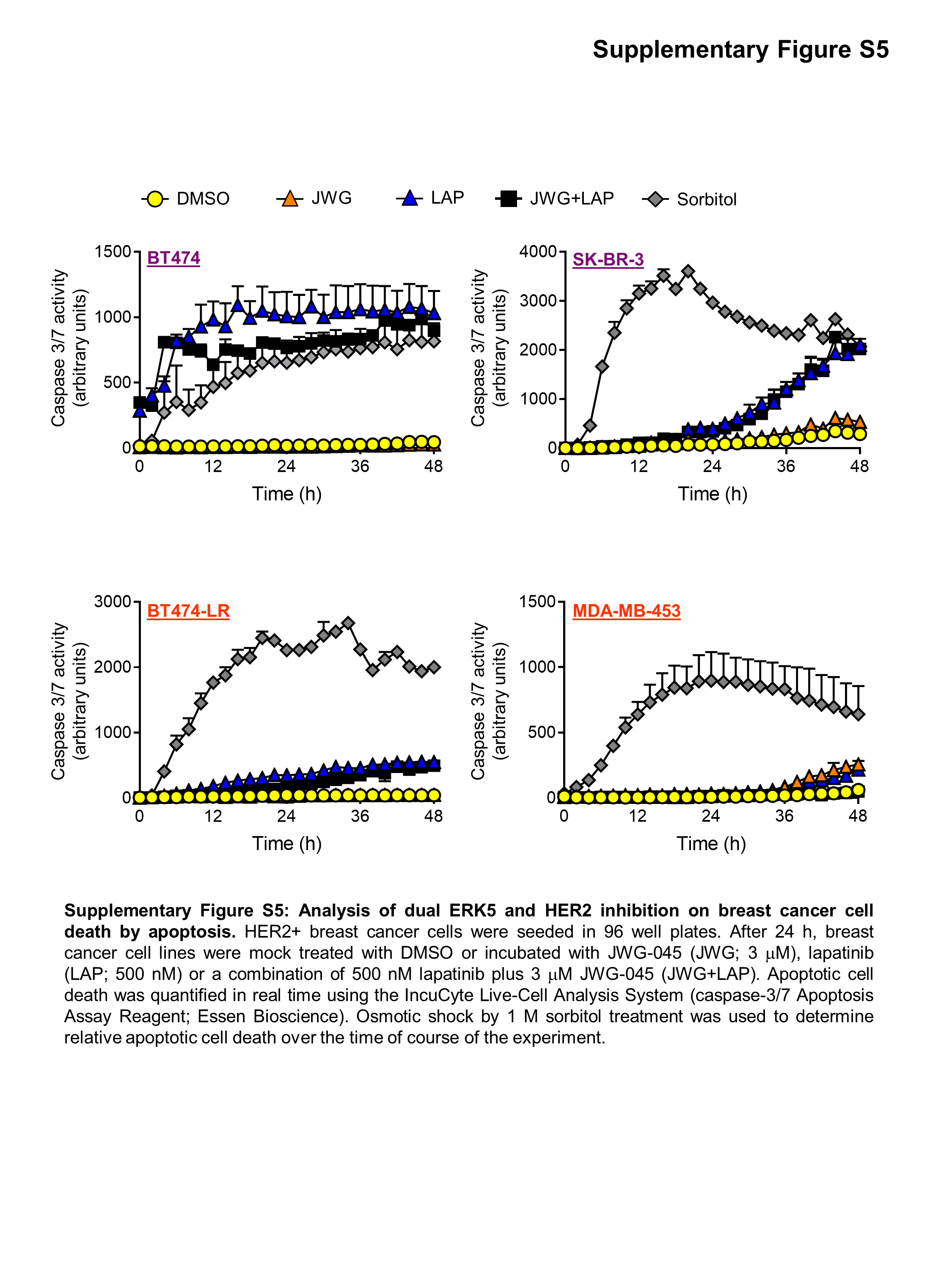

Supplement: Figure S5 — BT474, SK-BR-3, BT474-LR and MDA-MB-453 cell lines display distinct sensitivity to the apoptotic effect of 500 nM lapatinib. [file crc-21-0089-s05.png]

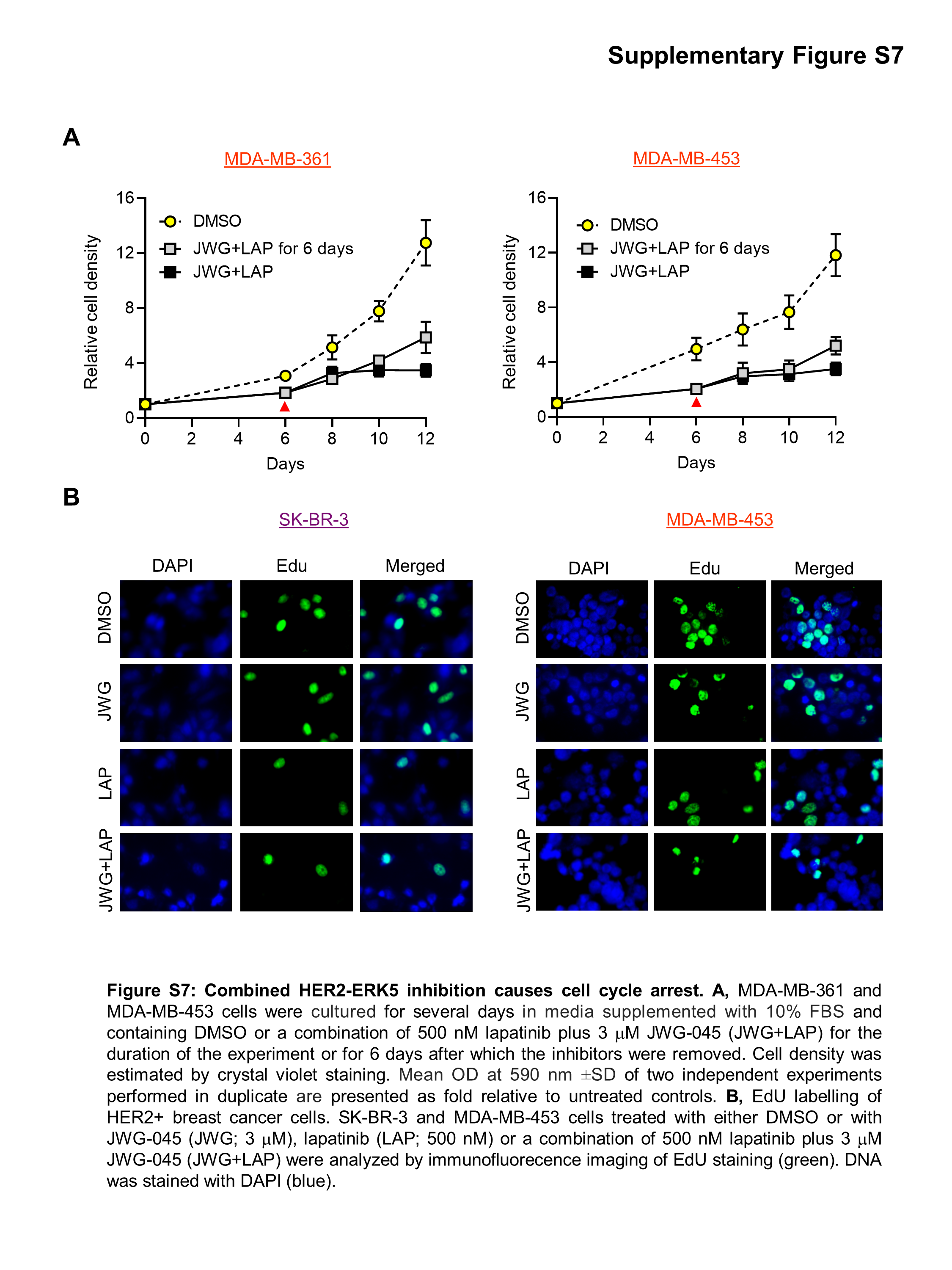

Supplement: Figure S7 — These data support the conclusion that ERK5 inhibtion enhances the cytostatic activity of lapatinib in resistance cells without increasing cytotoxicity. [file crc-21-0089-s07.png]

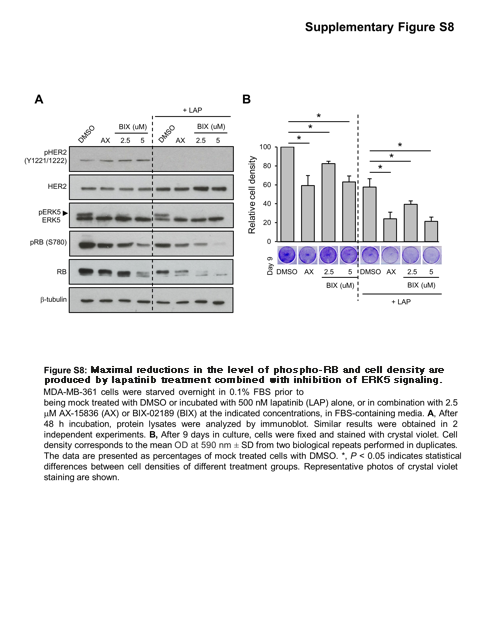

Supplement: Figure S8 — AX-15836 and BIX-02189 reproduce the inhibitory effect of JWG-045 on the level of phospho-RB and MDA-MB-361 cell density. [file crc-21-0089-s08.png]

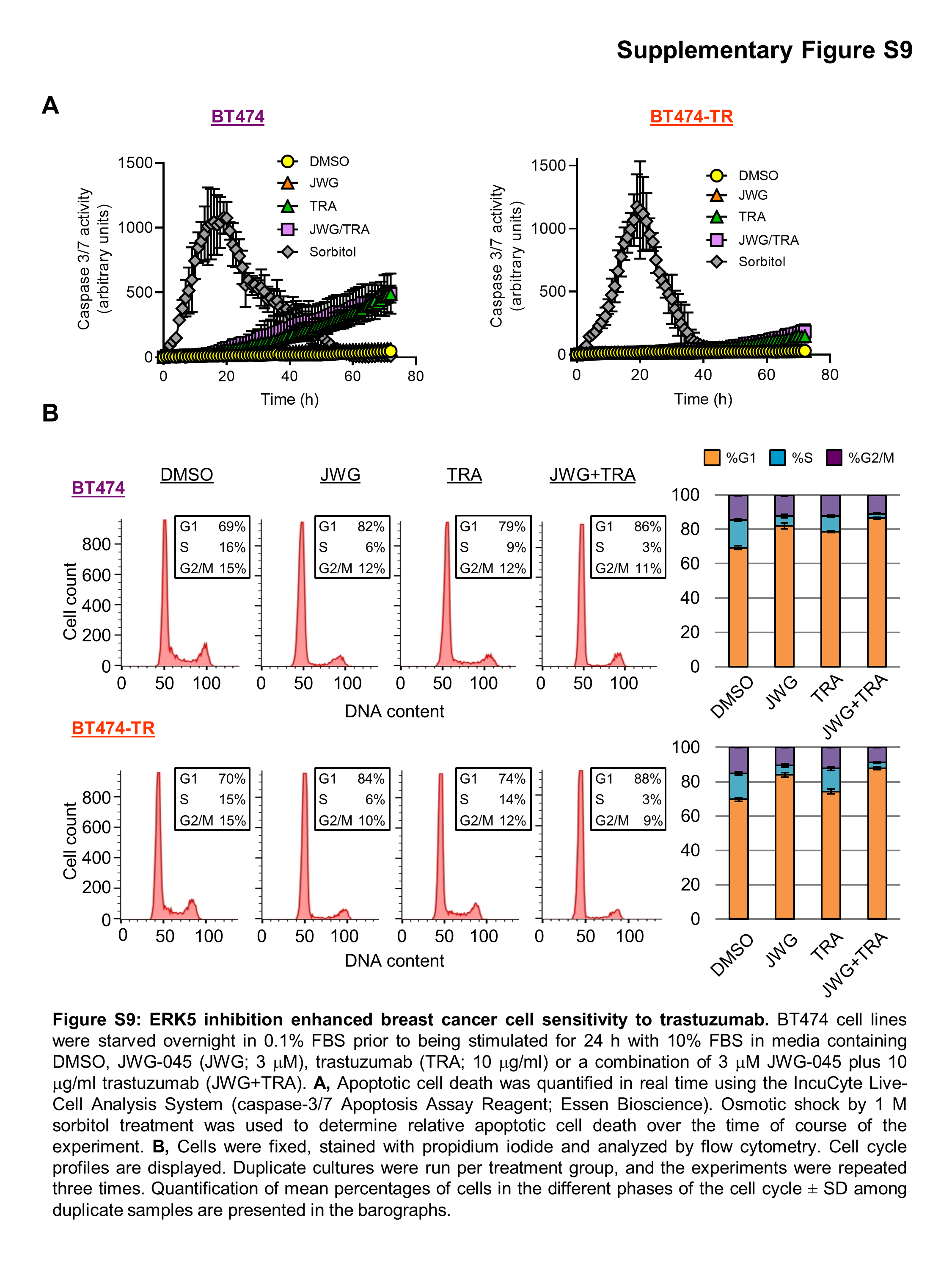

Supplement: Figure S9 — JWG-045 enhances the sensitivity of HER2+ breast cancer cells to trastuzumab through decreasing the proportion of cells in S phase, rather than increasing apoptotic cell death. [file crc-21-0089-s09.png]

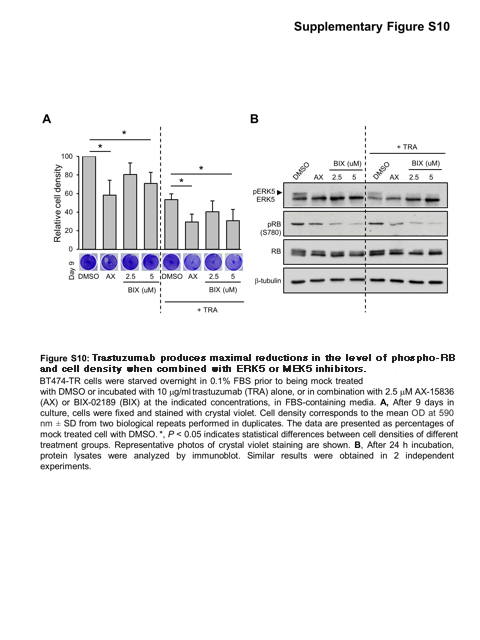

Supplement: Figure S10 — Inhibition of ERK5 by AX-15836 or MEK5 by BIX-02189 enhance HER2+ breast cancer cell sensitivity to trastuzumab through decreasing RB phosphorylation. [file crc-21-0089-s10.png]
