# Supplementary material for: Inhibiting ERK5 Overcomes Breast Cancer Resistance to Anti-HER2 Therapy By Targeting the G1–S Cell-Cycle Transition
Source: Cancer Res Commun. 2022 Mar 10;2(3):131–45. doi: 10.1158/2767-9764.CRC-21-0089 (PMC7613885; doi:10.1158/2767-9764.CRC-21-0089)
Supplement: Figure S3 — Lapatinib treatment inhibits tyrosine phosphorylation of HER1/2/3 in both sensitive and resistant breast cancer cells. [file crc-21-0089-s03.pdf]

Supplementary Figure S3

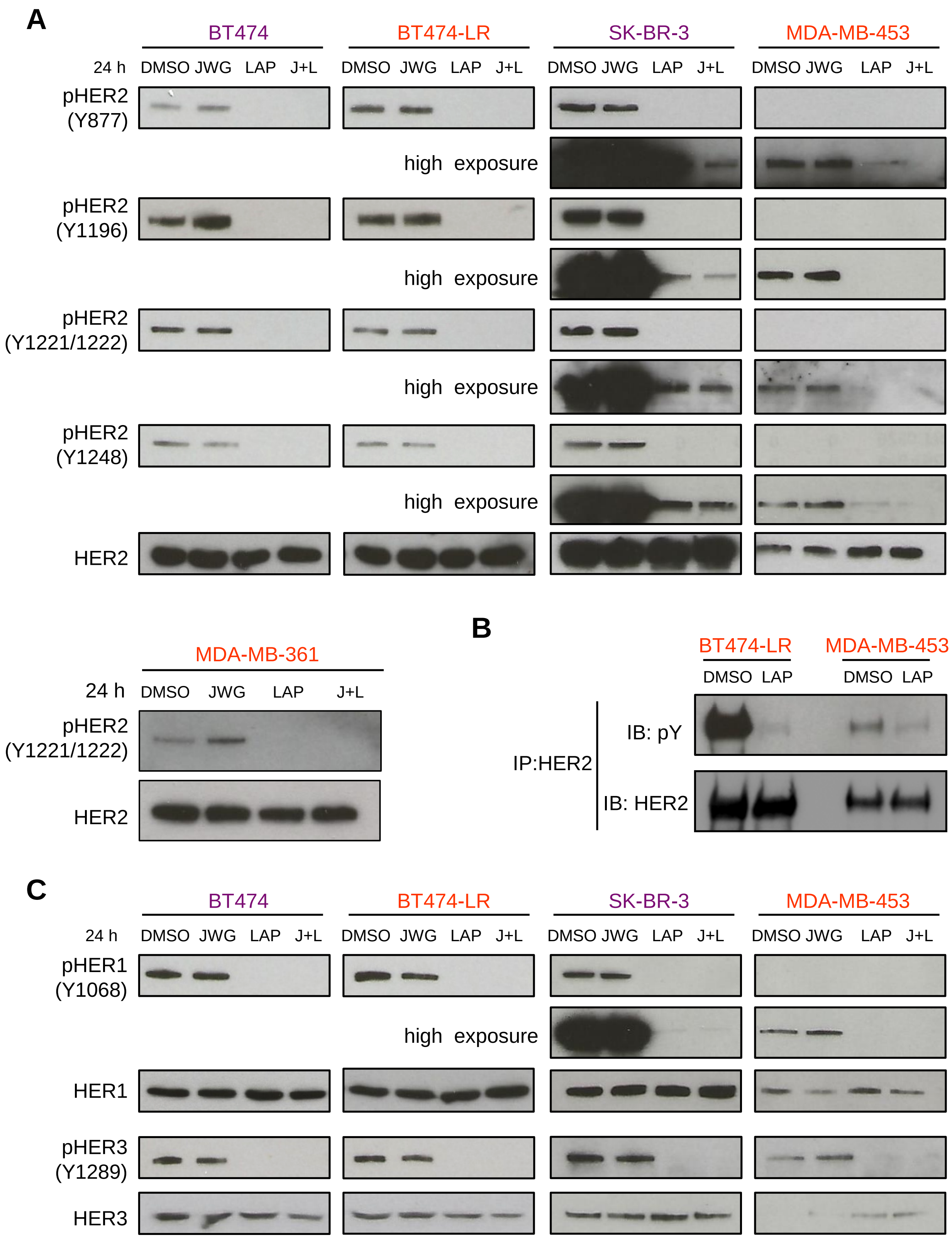

Supplementary Figure S3: Lapatinib blocks HER tyrosine kinase activity in sensitive and resistant cell lines. HER2+ breast cancer cells were starved overnight in 0.1% FBS, prior to being mock treated with DMSO, or incubated with JWG-045 (JWG, 3 mM), lapatinib (LAP, 1 mM) or both inhibitors together (J+L), for 24 h in 10% FBS-containing media. **A** and **C,** Protein lysates were analyzed by immunoblot with the indicated antibodies from CST: pHER2 (Y877; #2241), pHER2 (Y1221/1222; #2243), pHER2 (Y1196; #6942), pHER2 (Y1248; #2247), HER2 (#2165), pHER1/EGFR (Y1068#2234), HER1/EGFR (#4267), pHER3 (Y1289#4791), HER3 (#12708). β-tubulin expression was used as loading control. Similar results were obtained in two independent experiments. **B,** HER2 was immunoprecipitated prior to being analyzed by immunoblot with an antibody to HER2 or to phosphotyrosine epitopes. For this experiment, protein lysates were extracted in Pierce™ IP Lysis Buffer (Thermo Fisher Scientific # 87787) instead of RIPA buffer.
