# Supplementary material for: Inhibiting ERK5 Overcomes Breast Cancer Resistance to Anti-HER2 Therapy By Targeting the G1–S Cell-Cycle Transition
Source: Cancer Res Commun. 2022 Mar 10;2(3):131–45. doi: 10.1158/2767-9764.CRC-21-0089 (PMC7613885; doi:10.1158/2767-9764.CRC-21-0089)
Supplement: Figure S6 — The effect of lapatinib and JWG-045 as single treatments was compared to that achieved in combination therapy on the cell cycle profile of sensitive and resistant breast cancer cell lines. [file crc-21-0089-s06.pdf]

Supplementary Figure S6

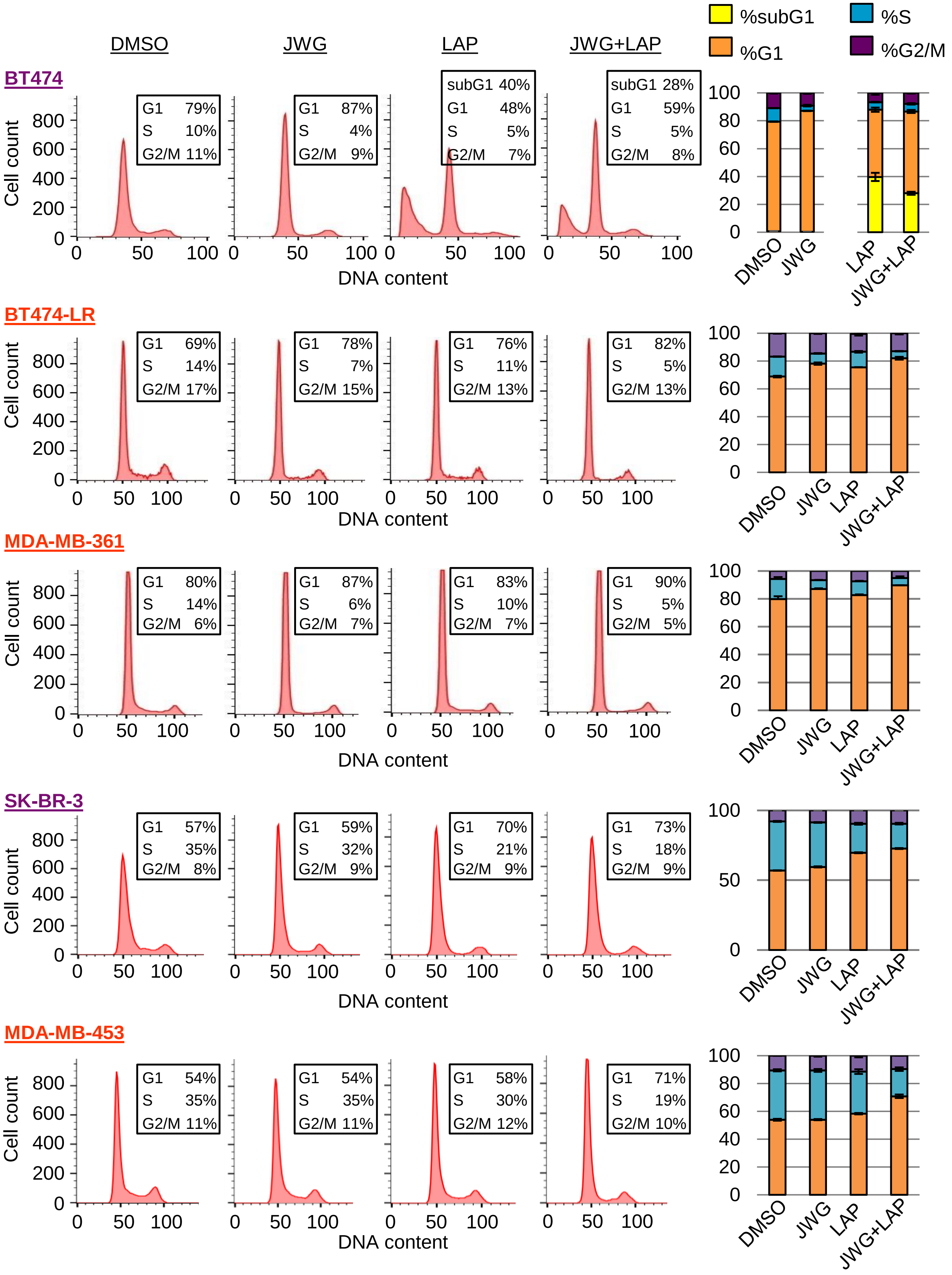

Supplementary Figure S6: Effect of combined ERK5 inhibition and lapatinib treatment on the cell cycle. Sub-confluent HER2+ breast cancer cells were starved overnight in 0.1% FBS prior to being stimulated for 24 h with 10% FBS in media containing DMSO, JWG-045 (JWG; 3 μM), lapatinib (LAP; 500 nM) or a combination of 3 μM JWG-045 plus 500 nM lapatinib (JWG+Lap). Cells were subsequently trypsinized, fixed in ice-cold 70% ethanol, stained with 500 mL propidium iodide (PI)/RNAse staining solution (Cell Signaling Technology # 4087) and analyzed by flow cytometry. Cell cycle profiles are displayed. Duplicate cultures were run per treatment group and the experiments were repeated two or three times. Quantification of mean percentages of cells in the different phases of the cell cycle ± SD among duplicate samples are presented in the barographs. Similar patterns were observed in at least two separated biological repeats. Different voltages were employed for analyzing the intensity of PI staining in BT474 cells treated and not treated with lapatinib. Interestingly, the robust G1 arrest caused by JWG-045 in the BT474 cell line counteracted the apoptotic effect of lapatinib, as indicated by a more moderate reduction in the percentage of cells in the subG1 phase. This observation is consistent with the idea that the most proliferative cells are also the most sensitive to the cytotoxic effects of anti-cancer agents.
