# Supplementary material for: Inhibiting ERK5 Overcomes Breast Cancer Resistance to Anti-HER2 Therapy By Targeting the G1–S Cell-Cycle Transition
Source: Cancer Res Commun. 2022 Mar 10;2(3):131–45. doi: 10.1158/2767-9764.CRC-21-0089 (PMC7613885; doi:10.1158/2767-9764.CRC-21-0089)
Supplement: Figure S11 — Combined ERK5 silencing plus lapatinib treatment caused a greater reduction in the percentage of breast cancer cells in S phase compared with lapatinib alone. [file crc-21-0089-s11.pdf]

# Supplementary Figure S11

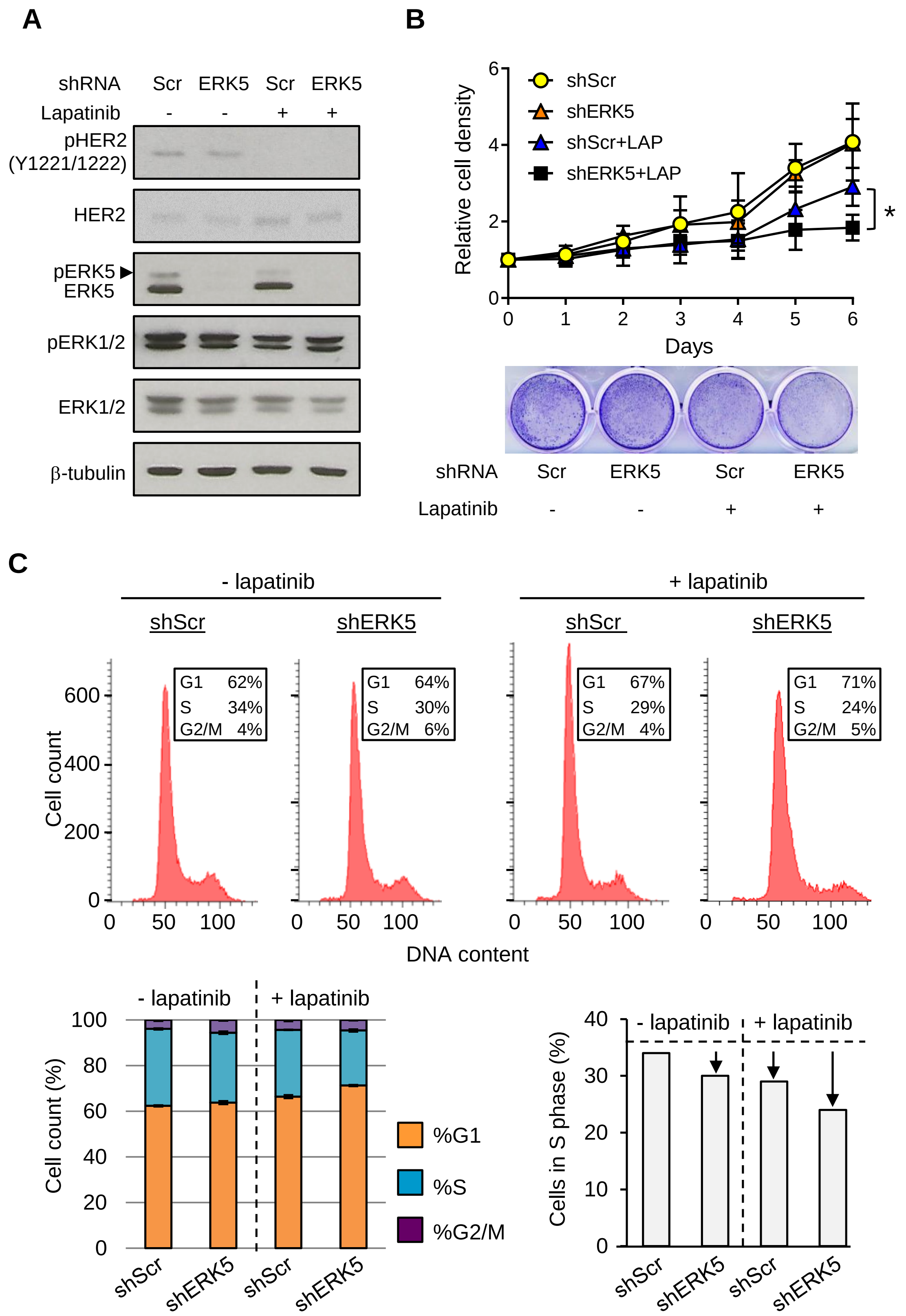

**Figure S11: ERK5 silencing in MDA-MB-543 cells. A,** MDA-MB-453 cells stably expressing shScr or shERK5 were starved overnight in 0.1% FBS prior to being mock treated with DMSO (-) or incubated with 1 mM lapatinib (+) for 24 h in 10% FBS containing media. Immunoblot analysis confirmed that shERK5 effectively silenced ERK5 expression. **B,** MDA-MB-453 cells were seeded at low density in 12 well plates. After 24 h, breast cancer cells were mock treated with DMSO (-) or incubated with lapatinib (LAP; 500 nM) for 6 days. Cell density was estimated by crystal violet staining. Mean OD at 590 nm ±SD of three independent experiments performed in duplicate are presented as fold relative to untreated controls. *, $P < 0.05$ indicates statistical differences between ERK5 knockdown plus lapatinib versus control shScr plus lapatinib at day 6. Representative photos of crystal violet staining of MDA-MB-453 cells at day 6 show that ERK5 silencing plus lapatinib reduced the number of MDA-MB-453 cells compared with lapatinib alone. **C,** MDA-MB-453 cells were fixed, stained with PI and analyzed by flow cytometry. Cell cycle profiles are displayed. Duplicate cultures were run per treatment group, and the experiments were repeated three times. Quantification of mean percentages of cells in the different phases of the cell cycle ± SD among duplicate samples and percentages of cells in S phase are presented in the barographs.
